# Butyric acid alleviates LPS-induced intestinal mucosal barrier damage by inhibiting the RhoA/ROCK2/MLCK signaling pathway in Caco2 cells

**DOI:** 10.1371/journal.pone.0316362

**Published:** 2024-12-26

**Authors:** Luqiong Liu, Tong Chen, Zhenrong Xie, Yongjin Zhang, Chenglu He, Yongkun Huang

**Affiliations:** 1 Kunming Medical University, Kunming, Yunnan, China; 2 Department of Pediatrics, the First Affiliated Hospital of Kunming Medical University, Kunming, Yunnan, China; 3 Centre for Experimental Studies and Research, the first Affiliated Hospital of Kunming Medical University, Kunming, Yunnan, China; 4 BioBank, the First Affiliated Hospital of Kunming Medical University, Kunming, Yunnan, China; 5 Department of Laboratory Medicine, the First Affiliated Hospital of Kunming Medical University, Kunming, Yunnan, China; Fukushima Medical University, JAPAN

## Abstract

Butyric acid (BA) can potentially enhance the function of the intestinal barrier. However, the mechanisms by which BA protects the intestinal mucosal barrier remain to be elucidated. Given that the Ras homolog gene family, member A (RhoA)/Rho-associated kinase 2 (ROCK2)/Myosin light chain kinase (MLCK) signaling pathway is crucial for maintaining the permeability of the intestinal epithelium, we further investigated whether BA exerts a protective effect on epithelial barrier function by inhibiting this pathway in LPS-induced Caco2 cells. First, we aimed to identify the optimal treatment time and concentration for BA and Lipopolysaccharide (LPS) through a CCK-8 assay. We subsequently measured Trans-epithelial electrical resistance (TEER), FITC-Dextran 4 kDa (FD-4) flux, and the mRNA expression of ZO-1, Occludin, RhoA, ROCK2, and MLCK, along their protein expression levels, and average fluorescence intensity following immunofluorescence staining. We then applied the ROCK2 inhibitor Y-27632 and reevaluated the TEER, FD-4 flux, and mRNA, and protein expression of ZO-1, Occludin, RhoA, ROCK2, and MLCK, as well as their distribution in Caco2 cells. The optimal treatment conditions were determined to be 0.2 mmol/L BA and 5 μg/mL LPS for 24 hours. Compared with LPS treatment alone, BA significantly mitigated the reduction in the TEER, decreased FD-4 flux permeability, increased the mRNA expression of ZO-1 and Occludin, and normalized the distribution of ZO-1 and Occludin in Caco2 cells. Furthermore, BA inhibited the expression of RhoA, ROCK2, and MLCK, and normalized their localization within Caco2 cells. Following treatment with Y-27632, the epithelial barrier function, along with the mRNA and protein expression and distribution of ZO-1 and Occludin were further normalized upon inhibition of the pathway. These findings contribute to a deeper understanding of the potential mechanisms through which BA attenuates LPS-induced impairment of the intestinal epithelial barrier.

## 1. Introduction

The epithelial barrier serves as the primary line of defense against various environmental factors, including physical, chemical, and immunological factors, and is widely accepted by experts in the field [1]. This barrier plays crucial roles in protecting host tissues from infections, environmental toxins, pollutants, and allergens, all of which can disrupt host homeostasis. As urbanization and economic development progress, humans continues to produce an increasing variety of hazardous substances, which pose a significant threat to the integrity of this protective barrier [2]. Furthermore, the relationships among the intestinal flora, microbial metabolites, and the intestinal barrier influence the occurrence and progression of various diseases [3]. The metabolites produced by the microbiota are vital for maintaining health [4]. In this context, dietary supplements, such as dietary fiber, can alter the levels of microbial metabolites and help restore the gut epithelial barrier, thereby promoting intestinal and systemic homeostasis, alleviating associated pathological conditions, and improving overall health outcomes.

Butyric acid (BA) is produced primarily by Firmicutes species, which include species from the families Lachnospiraceae, Ruminococcaceae, and Erysipelotrichaceae, through the glycolysis of dietary fiber. BA can be absorbed by colon epithelial cells, serving as the primary energy source for these cells. Additionally, it plays crucial roles in intestinal epithelial cells apoptosis, the maintenance of barrier integrity, the regulation of endocrine functions, and the immune response [5]. Furthermore, BA promotes the proliferation of colon mucosal epithelial cells and increases the tightness of epithelial cell connections at subtoxic concentrations [6]. Supplementation with short-chain fatty acids has demonstrated the potential to reduce intestinal permeability in various liver disease models [7]. Restoring the intestinal barrier is essential for treating both local and systemic diseases, irrespective of the underlying causes or consequences of intestinal epithelial injury [8]. Notably, sodium butyrate has been shown to alleviate LPS-induced diarrhea in mice by enriching beneficial bacteria and diminishing the levels of pathogens, thereby preserving epithelial barrier function [9].

Lipopolysaccharide (LPS) has been shown to reduce the expression of Zonula occludens-1 (ZO-1) and Occludin, thereby increasing the permeability of the intestinal mucosal barrier. Numerous studies have employed LPS to establish cellular or animal models of intestinal mucosal barrier damage [10]. Under standard culture conditions, Caco2 cells can spontaneously form a monolayer that exhibits apical and basal polarity, mirroring the differentiation characteristics of the intraluminal mucosal epithelium. However, following LPS treatment, trans-epithelial electrical resistance (TEER) decreases, while FITC-dextran permeability increases, and the expression levels of Occludin, ZO-1, and Claudin5 decrease [11]. Collectively, these findings indicate that BA may protect the intestinal barrier, in contrast with the detrimental effects of LPS.

Research has confirmed that ROCK and myosin light chain kinase (MLCK) play crucial roles in regulating tight junction proteins during intestinal inflammation and are implicated in the pathogenesis of irritable bowel syndrome and other related diseases [8]. The detrimental effects of Zearalenone on the intestinal barrier have been confirmed. It activates the RhoA/ROCK signaling pathway, leading to an increase in FITC-Dextran 4 kDa (FD-4) flux and a significant reduction in TEER. Furthermore, zearalenone marketedly upregulates MLCK and downregulates Occludin, claudin-1, ZO-1, and claudin-3 while also causing the mislocalization of ZO-1 in IPEC-J2 cells [12]. In a separate study involving bEnd cells, derived from three types of brain endothelial cells, an MLCK inhibitor was found to mitigate the downregulation of ZO-1 and further increase the permeability of the brain endothelial monolayer induced after OGD/R exposure [13]. Procyanidins have been shown to preserve normal intestinal barrier functions by inhibiting MLCK, similar to the effects of a MLCK inhibitor observed in a study involving acrylamide-induced Caco-2 cell monolayers [14]. Collectively, these findings suggest that the RhoA/ROCK2/MLCK pathway may play a vital role in the maintenance of epithelial monolayer integrity. Additionally, evidence indicates that butyrate can activate G protein-coupled receptors, including GPR41, GPR43, GPR109a, and GPR164, alter histone acetylation levels, promote crotonylation through histone acetylation, enhance interactions between PPARγ and various coactivators, and activate the human aryl hydrocarbon receptor [5]. However, no studies have specifically investigated the relationship between BA and the RhoA/ROCK2/MLCK pathway. Given the evidence presented, it is worthwhile to explore whether the RhoA/ROCK2/MLCK pathway is involved in the specific effect of BA on LPS-induced intestinal damage.

In this study, we aimed to investigate the optimal concentration and treatment time for BA and LPS to establish a reliable in vitro model of intestinal barrier dysfunction utilizing Caco2 cells. The effects of BA and LPS on barrier permeability and the expression of tight junction proteins were subsequently assessed. Furthermore, we explored the role of the RhoA/ROCK2/MLCK signaling pathway in protecting Caco2 cells from LPS-induced injury. These findings provide a critical theoretical foundation for elucidating the impact of BA on LPS-induced barrier damage and for developing novel clinical therapeutic strategies for intestinal injury. Additionally, the involvement of the RhoA/ROCK2/MLCK pathway in the protective effect of BA against LPS-induced intestinal damage merits further investigation.

## 2. Materials and methods

### 2.1 Cell culture

The human epithelial Caco-2 cells utilized in this study were obtained from Kunming Cell Bank, Typical Culture Preservation Committee, Chinese Academy of Sciences. These cells were cultured in Dulbecco’s Modified Eagle Medium (DMEM)/F12 (C11330500BT, Gibco) supplemented with 10% FBS (F8318-500 ml, Sigma) and 50 U/mL penicillin‒streptomycin. The cells were incubated in a humidified chamber containing 5% CO2 at 37°C (PHCbi, MCO-18AC, USP No. 6244103). Once the cell density reached 80–90%, the original culture medium was discarded, and the cells were washed twice with PBS and digested with 0.25% EDTA (25200056, Gibco, a pancreatic enzyme). After the cell density was adjusted, the cells were seeded in a cell culture plate and cultured until they stabilized and adhered to the surface.

### 2.2 Experimental groups and design

PBS (BL302A, Biosharp) was used to dissolve LPS (L4319, Sigma, USA), along with the diluents BA (107-92-6, DR.EHRENSTORFER) and Y-27632 (GC15712, GLPbio). These substances were further diluted in culture medium according to the appropriate experimental treatment and group. An equal volume of culture medium served as the control vehicle. Details regarding the cell experimental treatments and groups are presented in **Table 1**.

**Table 1:** Experimental treatments and groups.

Group	Treatment
**Effect of BA on cell viability**	
0, 0.05, 0.1, 0.2, 0.5, 1.0, 2.0, 5.0, 10.0 or 20.0 mmol/L BA	Treatment for 6 h, 12 h, 18 h, 24 h, 30 h or 36 h
**Effect of LPS on cell viability**	
LPS treatment for 18 h, 24 h or 36 h	0, 0.2, 0.5, 1.0, 2.0, 5.0, 10, 20, 50 or 100 μg/mL LPS
**Combined effects of BA and LPS on cell viability.**	
0, 2, 5, 10, 20 or 50 μg/mL LPS	0.2 mmol/L BA treatment for 24 h
**Effect of BA on LPS-induced Caco2 cells**	
Control group	Cultured in normal medium
BA group	Cultured with medium containing 0.2 mmol/L BA for 24 h
BL group	Cultured with medium containing 0.2 mmol/L BA and 5 μg/mL LPS for 24 h
LPS group	Cultured in medium containing 5 μg/mL LPS for 24 h
**Ability of BA to alleviates epithelial damage through the RhoA/ROCK2/MLCK signaling pathway in LPS-induced Caco2 cells**	
Control + Y-27632 group	Cultured with medium containing 10 μM Y-27632 for 24 h
BA + Y-27632 group	Cultured with medium containing 0.2 mmol/L BA and 10 μM Y-27643 for 24 h
BL + Y-27632 group	Cultured with medium containing 0.2 mmol/L BA, 5 μg/mL LPS and 10 μM Y-27643 for 24 h
LPS + Y-27632 group	Cultured with medium containing 5 μg/mL LPS and 10 μM Y-27643 for 24 h

### 2.3 Cell viability

To assess the viability of Caco2 cells, we performed the Cell Counting Kit-8 assay (GLPbio, USA) in a 96-well plate format. The cells were cultured according to the their experimental groups and 100 μL of serum-free medium supplemented with 10% CCK-8 was added. Following a 2-hour incubation at 37°C in the dark, the absorbance was measured at 450 nm using a microplate reader (Thermo Scientific, USA).

### 2.4 TEER measurements

TEER was measured 24 h after intervention using an ERS-2 epithelial voltohmmeter (Millicell-ER-2, Millipore MERS00002, USA). The cells were maintained at room temperature for 30 min to ensure stabilization. The electrical resistance was subsequently measured three consecutive times. The average value was then corrected to account for background resistance, and the values are expressed in units of Ω.cm^2^.

### 2.5 Measurement of the flux of the paracellular dye FD-4

FD-4 flux was employed to assess paracellular permeability. Caco-2 cells (1 × 10^4^/200 μL) were seeded into the upper chambers of a 24-well Transwell plate (3470, Costar, Corning), and 1.5 mL of fresh medium was added to the lower chambers. The medium was changed every other day to facilitate the formation of monolayers. Following treatment, the cells were incubated for 24 h in the upper chambers. The medium was subsequently replaced with serum-free medium containing FD-4 at a final concentration of 1 mg/mL. One milliliter of a serum-free medium was also added to the lower chambers, and the plate was incubated at 37°C for 2 h. After incubation, 100 μL of sample was extracted from the lower chambers and analyzed using a fluorescein enzyme-labeled instrument (Victor Nivo, PerkinElmer). The flux of FD-4 was calculated based on the standard curve derived from diluents of known concentrations.

### 2.6 Real-time quantitative PCR

Total RNA was extracted from Caco2 cells and the RNA concentration was quantified as follows.1). Total RNA was extracted via the TRIzol reagent (15596026; Invitrogen, Carlsbad, CA, USA), according to the manufacturer’s instructions. 2). The concentration and purity of the extracted RNA were assessed via an ultramicro spectrophotometer (NanoDrop 2000/2000c Spectrophotometer, ThermoScientific). 3). cDNA was synthesized using the RevertAid First Strand cDNA Kit (91258333, Thermo Scientific). Two micrograms of total RNA was used as a template for gene amplification apparatus (Thermal Cycler T960, Heal Force). 4). Real-time quantitative PCR was conducted using cDNA as the template and SYBR Green fluorescent dye (D7262, Beyotime) on a 7500 fast real-time PCR system (QuantStudioTM5, Life Technologies Holding Pte Ltd). 5). A 20 μL mixture, consisting of 2 μL of cDNA template, 6.0 μL of enzyme-free water, 10 μL of SYBR premixed fluorescence quantitative PCR reagent, and 1.0 μL of upstream and 1.0 μL of downstream primers. 6). The reaction conditions were as follows: predenaturation at 95°C for 2 min; PCR reaction stage: denaturation at 95°C for 15 sec; annealing at 60°C for 10 sec; extension at 72°C for 20 sec; and denaturation, annealing, and extension for a total of 40 cycles. The following conditions were used to obtain melting curves: 95°C for 15 s, 60°C for 60 s, and 95°C for 15 s. 7). The primer sets utilized are listed in **Table 2**. With GAPDH was used as a control and mRNA levels were calculated using the 2^-ΔΔCt^ formula. 8). Relative mRNA expression levels were normalized and are expressed as the fold change relative to the expression level in the control group.

**Table 2:** Primers for RT-qPCR.

Genes	Primers for RT‒qPCR	Reverse sequence (5′–3′)
GAPDH	CATGAGAAGTATGACAACAGCCT	AGTCCTTCCACGATACCAAAGT
ZO-1	ACCAGTAAGTCGTCCTGATCC	TCGGCCAAATCTTCTCACTCC
Occludin	ACAAGCGGTTTTATCCAGAGTC	GTCATCCACAGGCGAAGTTAAT
RhoA	AAGAGGCTGGACTCGGATTCGT	CCACAGGCTCCATCACCAACAAT
ROCK2	TCCCGATAACCACCCCTCTT	CCAAGGAATTTAAGCCATCCACT
MLCK	CCCGAGGTTGTCTGGTTCAAA	GCAGGTGTACTTGGCATCGT

### 2.7 Immunofluorescence staining and measurement of the average fluorescence intensity

Caco2 cells were fixed with 4% paraformaldehyde (BL539A, Biosharp) for 10 min. Following fixation, the cells were washed three times for 5 min each with PBS. The samples were then permeabilized with 1% Triton X-100 (T8200, Solarbio, China) for 10 min, and subsequently blocked with 10% goat serum (SL038, Solarbio) at 37°C. The cells were incubated overnight at 4°C with rabbit primary antibodies, against ZO-1 (ab96587, 1:500, Abcam), Occludin (27260-1-AP, 1:500, Proteintech), RhoA (10749-1-AP, 1:500, Proteintech), ROCK2 (20248-1-AP, 1:100, Proteintech, China), and MLCK (21642-1-AP, 1:100, Proteintech). After incubation, the cells were washed three times with PBS and then incubated at room temperature with fluorophore-labeled secondary antibodies (ab150081, 1:1000, Abcam) for 1 h. Finally, DAPI (P0131, Beyotime, China) was used to stain the nuclei in the dark. Images were captured via a **fluorescence microscope** (N-SIM/C2si, Nikon, Japan). At least six representative images **(1024 × 1024 pixels, 3 × 12 bits)** were captured for each group. The fluorescence intensities of the target proteins and DAPI in the selected six regions from each group were quantified using **Image J**. The specific operation process is as follows: ImageJ.exe →→ File →→ Open →→ Image →→ Color →→ OK →→ Image →→ Color →→ Split Channels (blue (DAPI) and green (ZO-1, Occludin, RhoA, ROCK2, and MLCK), do the following for each picture with two colors separately) →→ Image →→ Adjust →→ Threshold (Use the default threshold) →→ Apply →→ Image →→ Auto Threshold →→ Try all →→ OK→→ Default →→ Threshold →→ Default (If the threshold set by the default algorithm does not meet the requirements, another algorithm is needed.) →→ Apply →→ Analyze →→ Set measurements (Area, Mean gray value, Standard deviation, Integrated density, Limit to threshold, Display label) →→ OK →→ Analyze →→ Measure →→ Results →→ File →→ Save as →→ Results.csv →→Data analysis. The mean gray value was calculated as the average fluorescence intensity. The respective ratios of the fluorescence intensity of the target proteins (ZO-1, Occludin, RhoA, ROCK2, and MLCK) to the homologous DAPI fluorescence intensity were calculated. The average ratio of target protein fluorescence intensity to DAPI fluorescence intensity for the six regions of the control or CI groups was subsequently determined. Finally, the relative average fluorescence intensity of the target proteins was expressed as the ratio of the fluorescence intensity of target proteins to that of DAPI in each group and was normalized to the average ratio of the fluorescence intensity of the target proteins to the fluorescence intensity of DAPI in the control or CI group. Data analysis and visualization were performed via **GraphPad Prism 9.0 software**.

### 2.8 Statistical analysis

The data were collected, analyzed, and visualized via GraphPad Prism 9.0 (GraphPad Software, USA). Measurement data, which conforming to a normal distribution, are presented as the mean ± standard deviation (SD). A factorial design analysis was conducted, followed by Tukey’s multiple comparison test. A *P*-value of less than 0.05 was considered to indicate statistical significance.

## 3. Results

### 3.1 Optimal concentration and treatment time for BA and LPS in Caco2 cells

**Fig 1:**
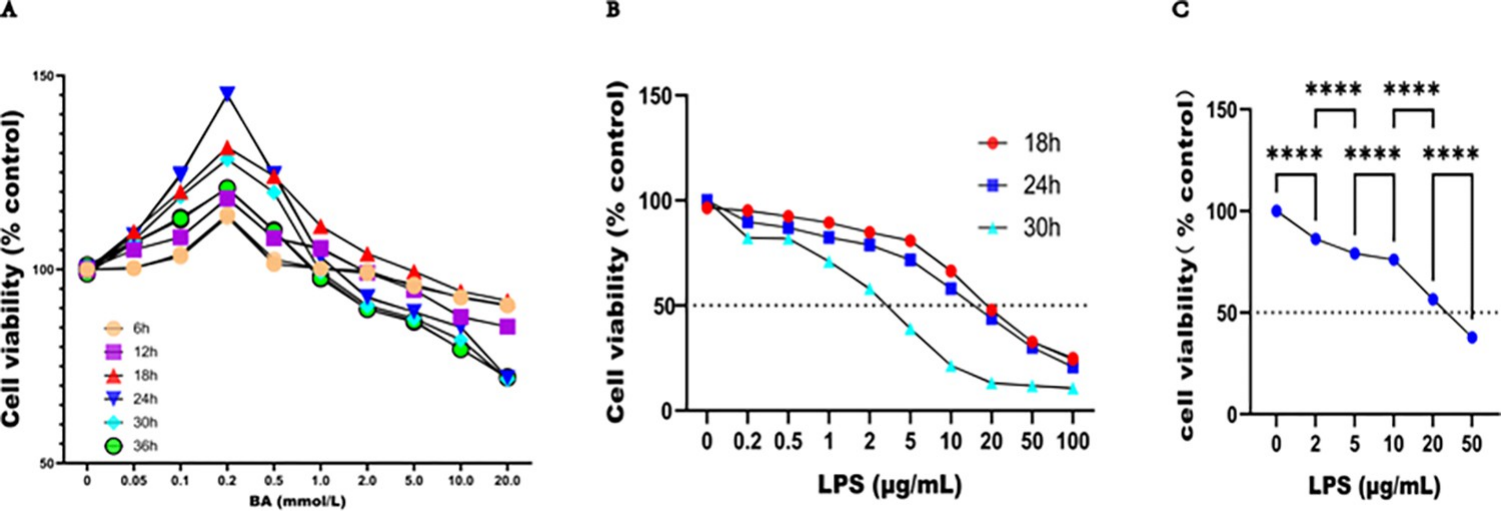
The optimal concentrations and treatment durations for BA and LPS in Caco2 cells. **(A)** The viability of Caco2 cells was assessed using the CCK8 assays after treatment with various concentrations of BA for 0 to 36 h. (**B)** Cell viability following treatment with different concentrations of LPS for 18 to 30 h was also evaluated using the CCK8 assay. (**C)** The viability of Caco2 cells was determined by the CCK8 assays following treatment with 0.2 mmol/L BA combined with different concentrations of LPS for 24 h. **** denotes < 0.0001.

**As shown in Fig 1A**, Caco2 cells were treated with various concentrations of BA for durations ranging from 0 to 36 h. Cell viability, which was determined by the CCK8 assay, was greater in the group with 0.2 mmol/L BA group than in the groups treated with the other concentrations of BA. This effect was most pronounced after treatment for 24 h. In **Fig 1B**, following treatment with different concentrations of LPS for 18,24 or 30 h, cell viability gradually decreased as the concentration of LPS increased. Notably, cell viability decreased rapidly after treatment with LPS for 24 h. Based on these findings, a final concentration of 0.2 mmol/L BA and various concentrations of LPS were coadministered to Caco2 cells for 24 h. **Fig 1C** shows that the protective effect of BA remained relatively stable when the LPS concentration ranged from 2 to 10 μg/mL. However, when the LPS concentration exceeded 10 μg/mL, cell viability decreased sharply. Furthermore, as shown in **Fig 1B**, the IC50 of LPS significantly increased after treatment with BA for 24 h (14.70 vs 29.11) μg/mL, indicating that BA increased the viability of Caco2 cells induced by LPS. Consequently, in subsequent experiments, cells were treated with 0.2 mmol/L BA and 5 μg/mL for a 24 h to establish an in vitro cell model.

### 3.2 BA alleviated the decrease in the integrity and increase in the permeability of the epithelial barrier injury induced by LPS in Caco2 cell monolayers

**Fig 2:**
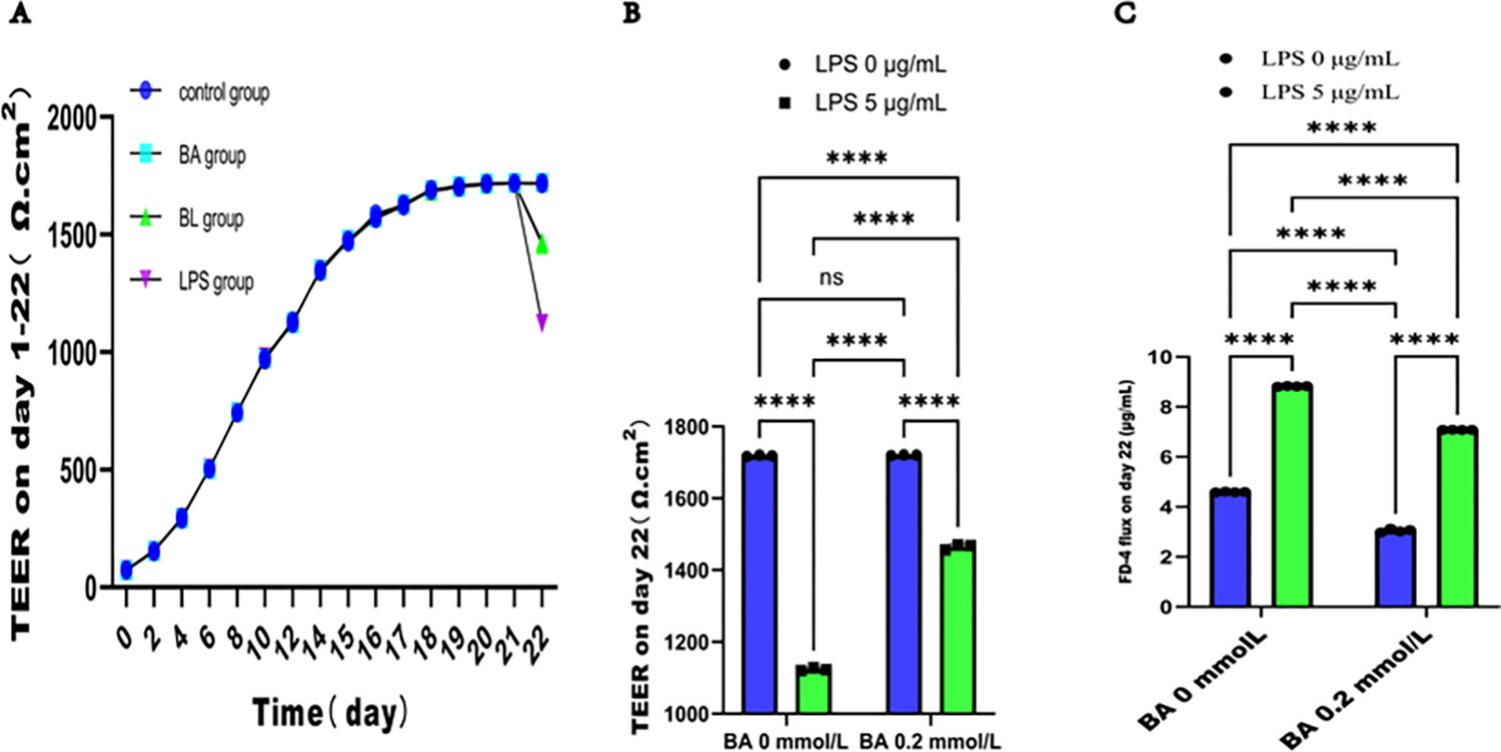
BA attenuated the decrease in the integrity and increase in the permeability of the epithelial barrier injury induced by LPS in Caco2 cell monolayers. (**A)** Changes in TEER with increasing culture time on days 1–22. (**B)** BA alleviated the LPS-induced decrease in TEER in Caco2 cells after treatment for 24 h. (**C)** BA alleviated the LPS-induced increase in FD-4 flux in Caco2 cells on day 22.

We evaluated the impact of BA on epithelial barrier function by measuring TEER and FD-4 flux. The TEER of Caco2 cell monolayers gradually increased over the culture period. As illustrated in **Fig 2A**, the TEER was stable and reached its peak on day 21 in all groups. Then, BA and LPS were added to the Caco2 cells according to the experimental protocol. After 24 h, the TEER was calculated again, and the concentration of FD-4 flux was measured. As shown in **Fig** 2B, no significant difference in TEER was observed between the BA group and the control group (1718.000 ± 2.000 Ω^.^cm^2^ vs. 1720.000 ± 1.000 Ω^.^cm^2^, *P* > 0.05). In contrast, compared with those in the control group, the TEER values in the BL group (1464.667 ± 7.572 Ω^.^cm^2^) and the LPS group (1123.667 ± 4.041 Ω^.^cm^2^) sharply decreased (*P* < 0.0001). The decrease was more obvious in the LPS group than in the BL group (*P* < 0.0001). Furthermore, **Fig 2C** showed that FD-4 flux on day 22 was decreased in the BA group compared with the control group, whereas it was increased in both the BL and the LPS groups. In contrast, FD-4 flux was significantly greater in the LPS group than in the BL group. Collectively, these results indicate that BA can mitigate the LPS-induced reduction in TEER and the corresponding increase in FD-4 flux, thereby increasing the integrity and decreasing the permeability of the epithelial barrier.

### 3.3 BA attenuated the decrease in the expression and normalized the distribution of ZO-1 and Occludin in LPS-induced Caco2 cells

**Fig 3:**
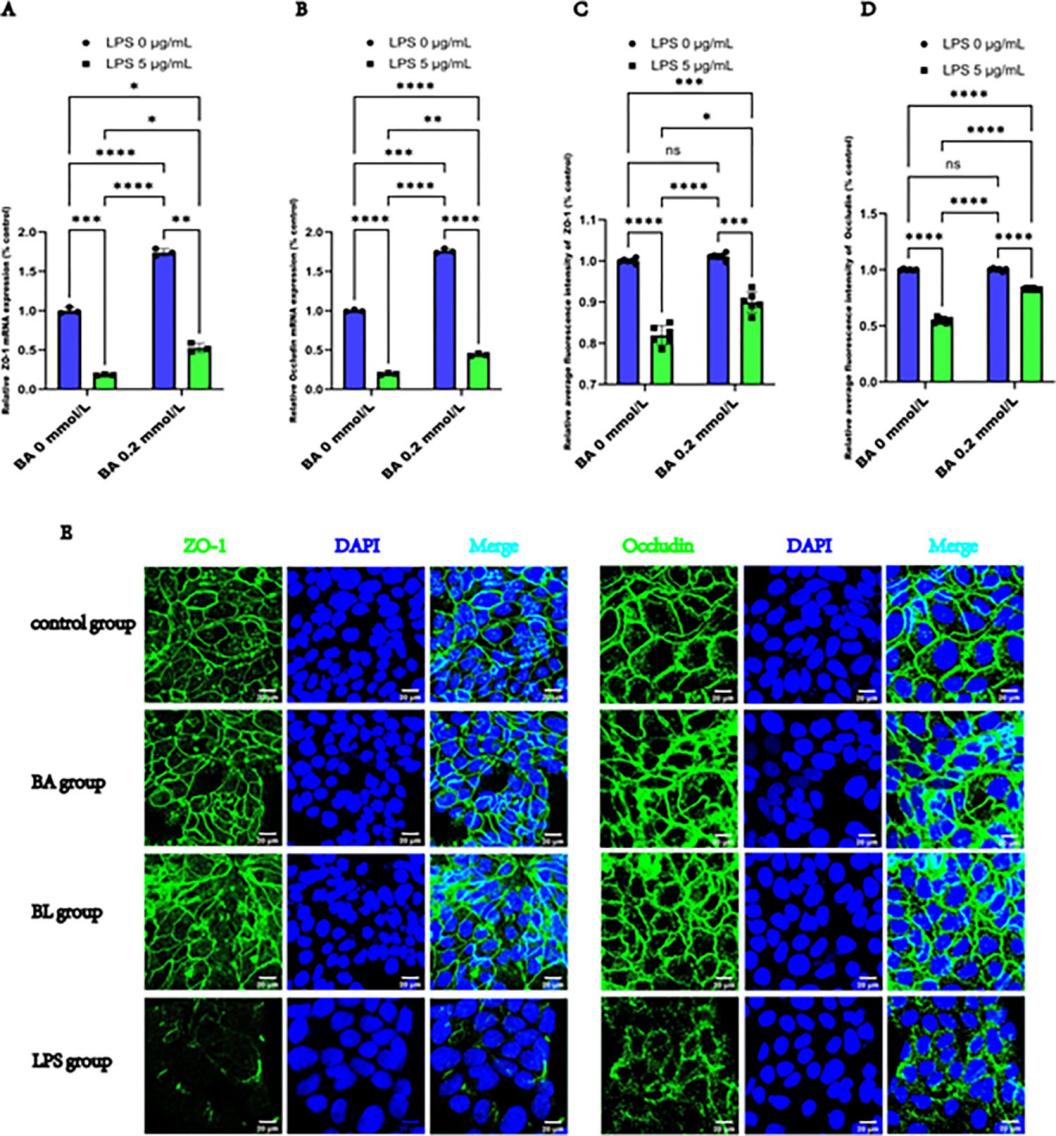
BA attenuated the decrease in the expression of ZO-1 and Occludin induced by LPS in Caco2 cells. **(A)** Relative mRNA expression levels of ZO-1. (**B)** Relative mRNA expression levels of Occludin. (**C)** Average relative fluorescence intensity of ZO-1 in Caco2 cells. (**D)** Average relative fluorescence intensity of Occludin. (**E)** Representative images of immunofluorescence staining for labeling ZO-1 and Occludin (antibodies, green) and nuclei (DAPI, blue) (scale bar  =  20 μm). The values are expressed as the means ± SDs and were analyzed according to the variance of the factorial design. *, **, *** and **** denote *p* < 0.05, < 0.01, < 0.001 and < 0.0001, respectively; ns  =  not significant.

To investigate the effects of BA on epithelial function, we evaluated the changes in ZO-1 and Occludin expression using RT-qPCR and immunofluorescence staining. Our findings revealed that ZO-1, and Occludin mRNA expression was significantly downregulated in the LPS-induced group compared with the control group. Conversely, BA increased ZO-1 and Occludin mRNA expression and alleviated the decrease in ZO-1 and Occludin mRNA expression in LPS-induced Caco2 cells, as illustrated in **Fig 3A and 3B**. Semiquantitative analysis of the average immunofluorescence intensity revealed that the expression levels of ZO-1 and Occludin were comparable between the control and BA groups. Notably, the expression of ZO-1 and Occludin was significantly downregulated in the LPS-induced group compared with the control group. The expression of ZO-1 and Occludin was lower in the LPS group than in the BL group in **Fig 3C and 3D**. We also noted that ZO-1 and Occludin formed a distinct, continuous, and organized network around the plasma membrane, forming cell-cell junctions, in both the control and BA groups, although this network was particularly pronounced in the BA group. After 24 h of LPS exposure, Caco2 cells exhibited depletion of ZO-1 and Occludin at several sites, with the BL group and especially the LPS group displaying a loose and discontinuous network, that appeared serrated or notched and fissured in the BL and LPS groups, especially in the LPS group. Additionally, cytoplasmic accumulation of ZO-1 and Occludin was observed in certain regions in the BL and LPS groups. However, BA alleviated the alterations in the distribution of ZO-1 and Occludin in Caco2 cells, as depicted in **Fig 3E**. Our results demonstrate that BA effectively reverses LPS-induced abnormalities in the expression and localization of ZO-1 and Occludin, highlighting the importance of tight junction integrity for the protective effects of BA.

### 3.4 BA inhibited the RhoA/ROCK2/MLCK signaling pathway in LPS-induced Caco2 cells

**Fig 4:**
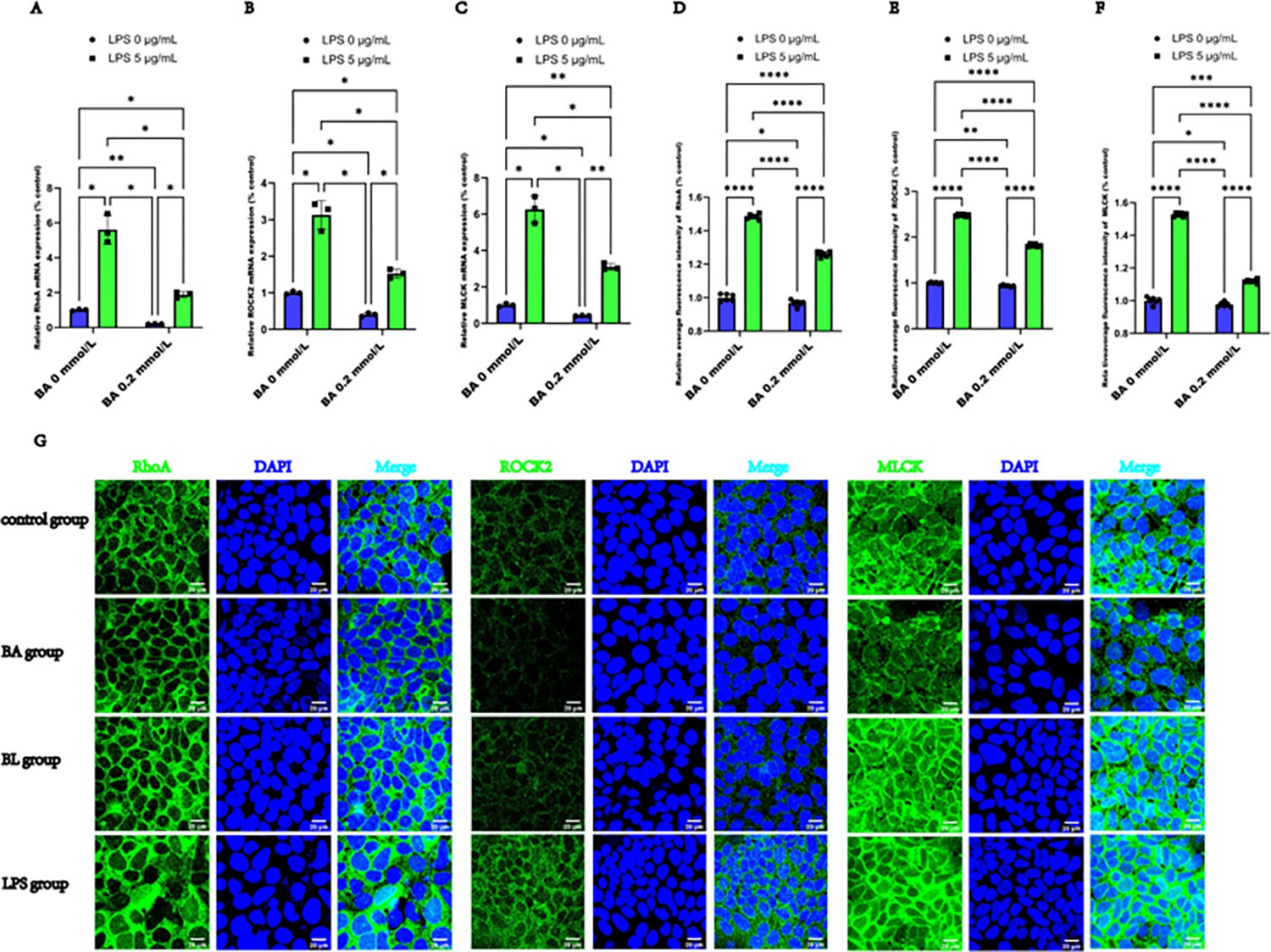
Inhibitory effects of BA on the RhoA/ROCK2/MLCK signaling pathway in LPS-induced Caco2 cells. (A) Relative mRNA expression of RhoA following exposure to LPS and BA in Caco2 cells. (B) Relative mRNA expression of ROCK2. (C) Relative mRNA expression of MLCK. (D) Average relative immunofluorescence intensity of RhoA after exposure to LPS and BA in Caco2 cells. (E) Average relative immunofluorescence intensity of ROCK2. (F) Average relative immunofluorescence intensity of MLCK in Caco2 cells after exposure to LPS and BA. (G) Representative images of immunofluorescence staining for RhoA, ROCK2, and MLCK (antibodies, green) and nuclei (DAPI, blue) (scale bar  =  20 μm). The values are expressed as the means ± SDs and were analyzed according to the variance of the factorial design. *, **, *** and **** denote *p* < 0.05, < 0.01, < 0.001 and< 0.0001, respectively.

Given that RhoA/ROCK2 and MLCK are crucial for regulating epithelial tight junctions and paracellular leakage-related pathways [15], we further investigated the activity of the RhoA/ROCK2/MLCK pathway in LPS-induced Caco2 cells. Our results revealed that after 24 h of treatment, the mRNA levels of RhoA, ROCK2, and MLCK were increased in both the BL and LPS groups, compared with the control group, but decreased in the BA group. However, BA treatment partly reversed the changes in the expression of these mRNAs in **Fig 4A–4C**. Semiquantitative analysis of the average immunofluorescence intensity revealed that the relative expression of RhoA, ROCK2, and MLCK was increased in the BL and LPS groups, compared with the control group, but decreased in the BA group. BA treatment partly reversed the increase in the expression of these proteins in the BL group compared with the LPS group in **Fig 4D–4F**. Immunofluorescence staining in **Fig 4G** revealed that RhoA was predominantly localized in the cell membrane and cytoplasm in both the control and BA groups, while its expression increased significantly after LPS stimulation and moved to the nucleus. ROCK2 was distributed mainly in the cytoplasm, whereas its expression expanded significantly in the cytoplasm and moved to the membrane after LPS stimulation. In the control and BA group, MLCK was localized mainly in the cytoplasm, however, its expression markedly increased in the cytoplasm following LPS exposure, and it was observed to be translocated to both the nucleus and membrane. Conversely, BA decreased the increased expression of these proteins and partially reversed their subcellular localization. Collectively, these data suggest that BA may improve the epithelial barrier function of Caco2 cells by inhibiting the RhoA/ROCK2/MLCK pathway.

### 3.5 Y-27632 treatment enhanced the protective effect of BA on LPS-induced Caco2 cells

To investigate the role of the RhoA/ROCK2/MLCK pathway in preventing LPS-induced epithelial injury to Caco2 cells, cells were treated with an inhibitor of ROCK2 called Y-27632, while excluding BA or LPS. We then assessed the integrity and permeability of the Caco2 cell monolayer, as well as the expression of the tight junction proteins ZO-1, Occludin, RhoA, ROCK2, and MLCK, and their distribution within the Caco2 cells.

#### 3.5.1 Y-27632 treatment enhanced the protective effect of BA on the integrity and permeability of LPS-induced Caco2 cell monolayers

**Fig 5:**
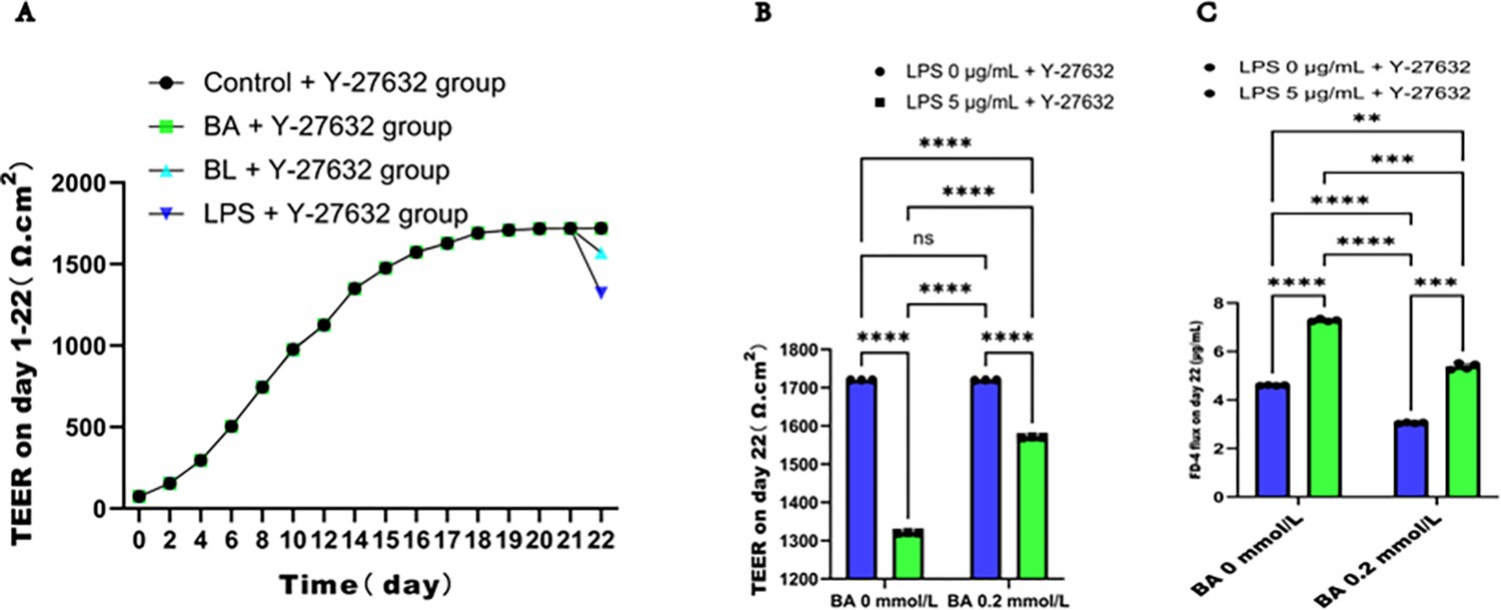
Y-27632 collaborated with BA to attenuate the increase in the integrity and decrease in the permeability of epithelial barrier injury induced by LPS in Caco2 monolayers. (**A)** Y-27632 collaborated with BA to attenuate the effect of LPS on TEER in Caco2 cells on days 1–22. (**B)** Y-27632 collaborated with BA to attenuate the effect of LPS on TEER in Caco2 cells on day 22. (**C**) Y-27632 collaborated with BA to alleviate the decrease in FD-4 flux in LPS-induced Caco2 cells on day 22. The values are expressed as the means ± SDs and were analyzed according to the variance of the factorial design. **, *** and ****denote *p* < 0.01, < 0.001 and < 0.0001, respectively; ns  =  not significant.

The TEER value of Caco2 monolayer cells gradually increased with prolonged culture time. As illustrated in **Fig 5A**, TEER stabilized and reached its peak on day 21 in all the experimental groups. Subsequently, BA or LPS and Y-27632 were added to the Caco2 cells according to the experimental design. After 24 h, TEER was recalculated and the concentration of FD-4 flux was measured. On day 22, the changes in TEER and FD-4 flux concentration were comparable to those observed in the absence of Y-27632. On day 22, as shown in **Fig 5B**, compared to that in the control + Y-27632 group (1720.000 ± 0.000 Ω^.^cm^2^), TEER was no significance (*P* > 0.05) in the BA + Y-27632 group (1719.667 ± 0.577 Ω^.^cm^2^). However, the TEER value significantly decreased in the BL + Y-27632 group (1570.000 ± 1.000 Ω^.^cm^2^) and the LPS + Y-27632 group (1320.000 ± 1.000 Ω^.^cm^2^) (*P* < 0.0001), and more obvious alterations were detected in the LPS + Y-27632 group (*P* < 0.0001). Compared with that in the BL + Y-27632 group, the TEER value in the LPS+ Y-27632 group was significantly lower(*P* < 0.0001). Moreover, Y-27632 mitigated the decrease in TEER caused by LPS. EER was decreased in both the BL + Y-27632 group and the LPS + Y-27632 group, with a more significant decrease in the latter. Compared with that in the BL + Y-27632 group, the TEER value in the LPS + Y-27632 group was significantly lower. However, Y-27632 alleviated the decrease in TEER caused by LPS. Additionally, **Fig 5C** found that the flux of FD-4, a dye, used to measure the permeability of the epithelial barrier, was increased in the BL + Y-27632 and LPS + Y-27632 groups, with a more significant increase in the latter. Conversely, compared with that in the control + Y-27632 group, the concentration of FD-4 in the BA + Y-27632 group decreased. Moreover, Y-27632 partially reversed the increase in the FD-4 concentration induced by LPS. Our results demonstrate that Y-27632, in conjunction with BA, partially reverses the LPS-induced decrease in epithelial barrier integrity and increase in epithelial barrier permeability of the in Caco2 cell monolayers.

#### 3.5.2 Y-27632 treatment enhanced the protective effect of BA on the expression and distribution of ZO-1 and Occludin in LPS-induced Caco2 cells

**Fig 6:**
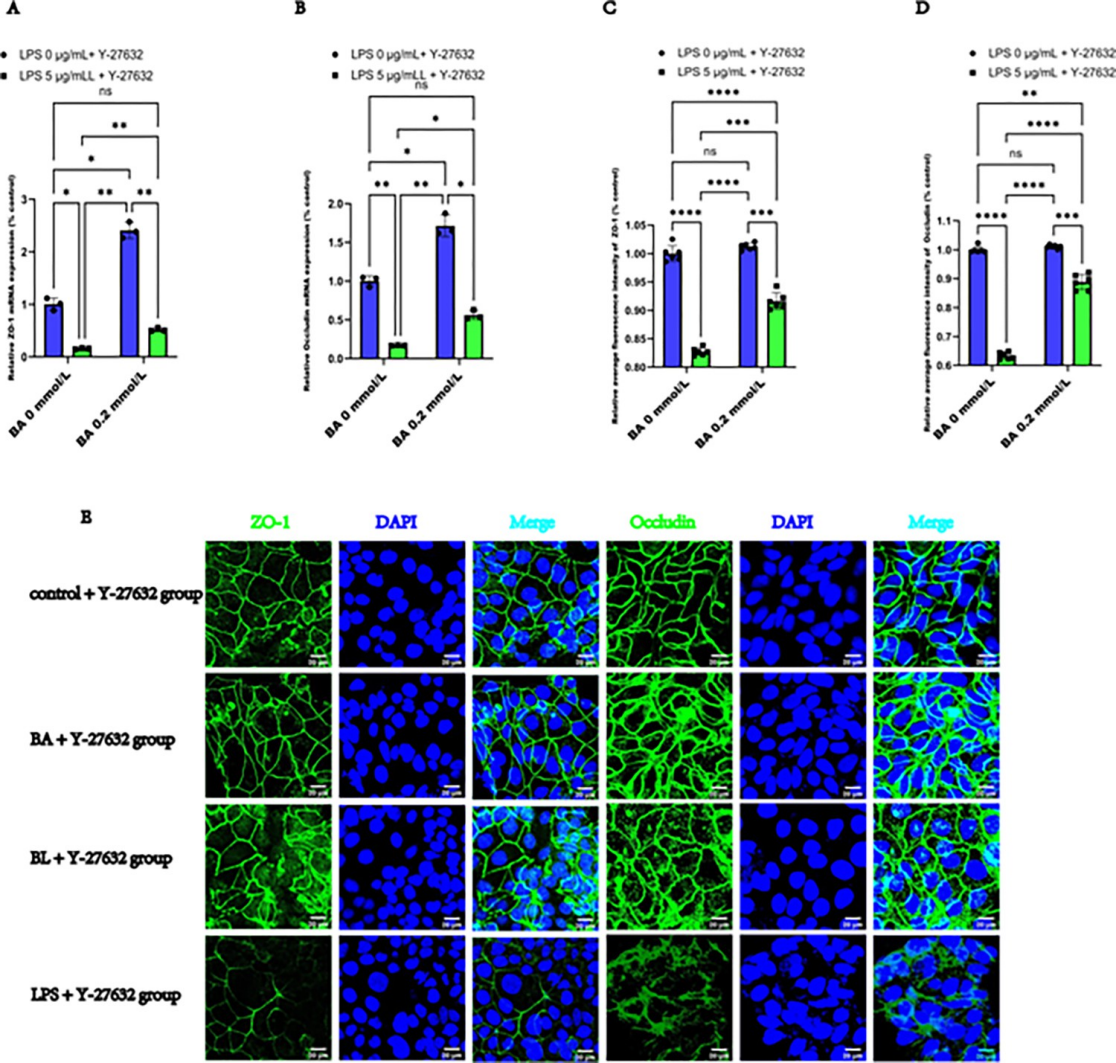
Y-27632 synergized with BA to alleviate the decrease in the expression of and abnormal localization of ZO-1 and Occludin in LPS-induced Caco2 cells. (A) Relative mRNA expressions of the mRNA levels of ZO-1 after exposure to LPS, BA, and Y-27632 for 24 h. (B) Relative mRNA expressions of Occludin. (C) Average relative fluorescence intensity of ZO-1 in Caco2 cells exposed to LPS, BA, and Y-27632 for 24 h. (D) Average relative fluorescence intensity of Occludin. (E) Representative images of immunofluorescence staining by labeling ZO-1 and Occludin (antibodies, green) and nuclei (DAPI, blue) (scale bar  =  20 μm) in Caco2 cells exposed to LPS, BA, and Y-27632 for 24 h. The values are expressed as the means ± SDs and were analyzed by variance of factorial design. *, **, *** and **** denote *p* < 0.05, < 0.01, < 0.001 and < 0.0001, respectively; ns  =  not significant.

In addition to influencing the integrity and permeability of LPS-induced Caco2 cell monolayers, Y-27632 and BA synergistically increased the expression of ZO-1 and Occludin, and normalized their localization in the Caco2 cells. As shown in **Fig 6A and 6B**, the mRNA levels of ZO-1 and Occludin were greater in the BA +Y-27632 group, and lower in the BL + Y-27632 group and the LPS + Y-27632 group than in the control + Y-27632 group. The mRNA levels of ZO-1 and Occludin in the BL + Y-27632 group were greater than those in the LPS + Y-27632 group. Moreover, semiquantitative analysis of the average relative immunofluorescence intensity revealed that the levels of ZO-1 and Occludin did not increase in the BA +Y-27632 group, but decreased in the BL + Y-27632 group and in the LPS + Y-27632 group compared with the control + Y-27632 group. The levels of ZO-1 and Occludin in the BL + Y-27632 group were greater than those in the LPS + Y-27632 group in **Fig 6C and 6D**. Furthermore, immunofluorescence staining showed that exposure to Y-27632 and BA led to increased expression of ZO-1 and Occludin on the cell membrane in LPS-induced Caco2 cells and normalized their distribution, and facilitated the formation of a clear, continuous, and organized network around the plasma membrane, constituting a cell‒cell junctions (**Fig 6E**). Collectively, these data demonstrate that Y-27632 synergizes with BA to alleviate the decreased in the expression and abnormal localization of ZO-1 and Occludin, indicating that the RhoA/ROCK2/MLCK signaling pathway may be involved in the protective effects of BA on tight junction proteins.

#### 3.5.3 Y-27632 synergized with BA to inhibit the RhoA/ROCK2/MLCK pathway in LPS-induced Caco2 cells

**Fig 7:**
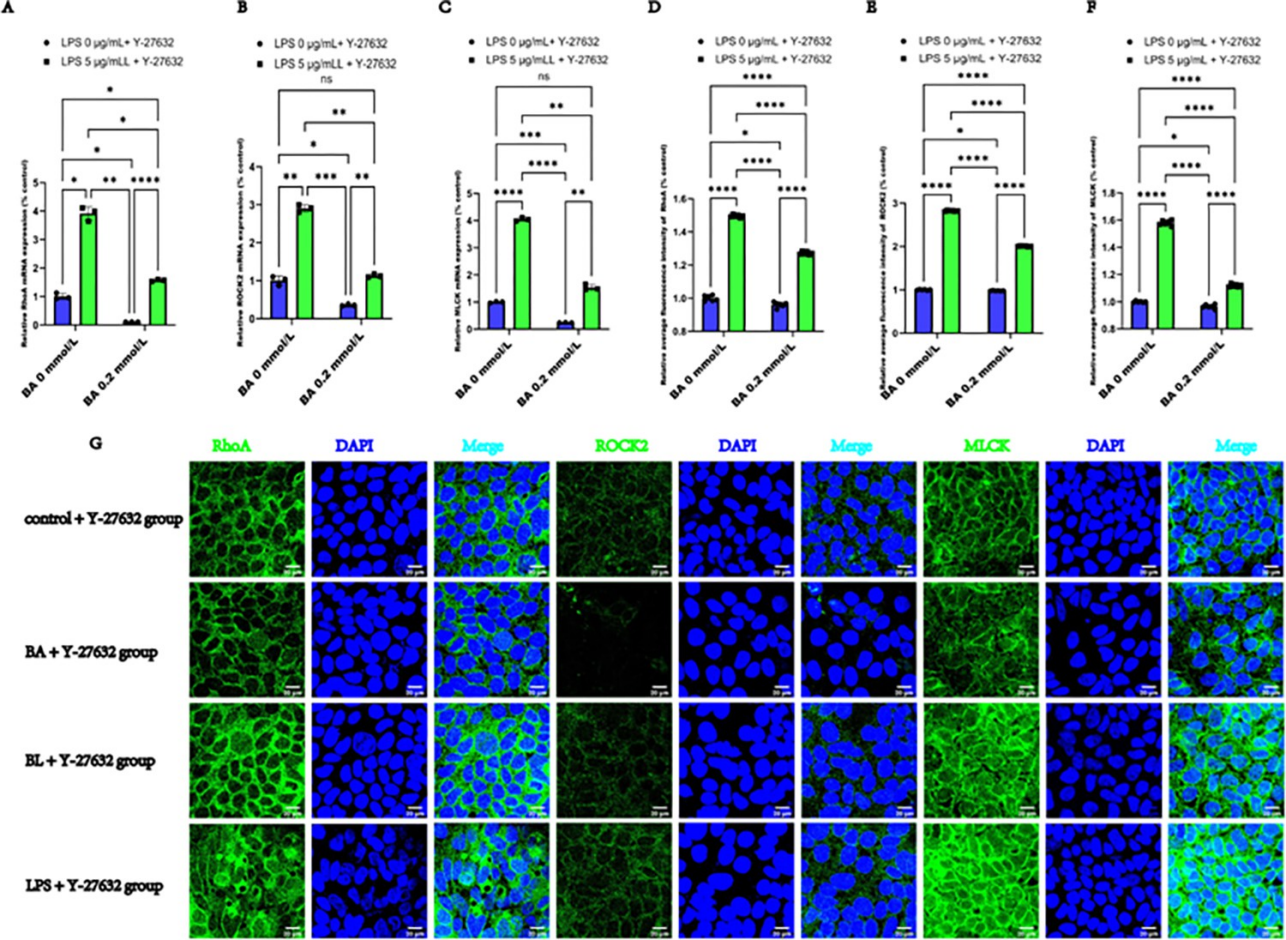
Y-27632 synergized with BA to inhibit the RhoA/ROCK2/MLCK signaling pathway in LPS-induced Caco2 cells. (**A)** Relative mRNA expressions of RhoA after exposure to LPS, BA, and Y-27632 in Caco2 cells for 24 h. (**B)** Relative mRNA expressions of ROCK2. (**C)** Relative mRNA expressions of MLCK. (**D)** Average relative immunofluorescence intensity of RhoA in Caco2 cells after exposure to LPS, BA, and Y-27632 for 24 h. (**E)** Average relative immunofluorescence intensity of ROCK2. (**F)** Average relative immunofluorescence intensity of MLCK. (**G)** Representative images of immunofluorescence staining by labeling RhoA, ROCK2, and MLCK (antibodies, green) and nuclei (DAPI, green) (scale bar  =  20 μm) in Caco2 cells after exposure to LPS, BA, and Y-27632 for 24 h The values are expressed as the means ± SDs and were analyzed according to the variance of the factorial design. *, **, *** and **** denote *p* < 0.05, < 0.01, < 0.001 and < 0.0001, respectively; ns  =  not significant.

Furthermore, the effects of the pathway of RhoA/ROCK2/MLCK pathway were investigated following the exposure of Caco2 cells to Y-27632. As illustrated in **Fig 7A–7C**, the mRNA levels of RhoA, ROCK2, and MLCK were lower in the BA + Y-27632 group than in the control + Y-27632 group. In the BL +Y-27632 group, the mRNA level of RhoA increased, whereas the mRNA levels of ROCK2 and MLCK did not increase. In the LPS + Y-27632 group, the mRNA levels of RhoA, ROCK2, and MLCK increased. The mRNA levels of RhoA, ROCK2, and MLCK in the BL + Y-27632 group were lower than those in the LPS + Y-27632 group. Moreover, as illustrated in **Fig 7D–7F,** semiquantitative analysis of average immunofluorescence intensities illustrated that the relative expression of RhoA, ROCK2, and MLCK was lower in the BA + Y-27632 group than in the control + Y-27632 group. In the BL +Y-27632 and LPS + Y-27632 groups, the average relative immunofluorescence intensity of RhoA, ROCK2, and MLCK increased. The average relative immunofluorescence intensities of RhoA, ROCK2, and MLCK in the BL + Y-27632 group were lower than those in the LPS + Y-27632 group. In addition, the changes in the mRNA and protein expression of RhoA in **Fig 7** were consistent with those shown in **Fig 4**, whereas the expression of ROCK2 and MLCK showed obvious changes. Immunofluorescence staining in **Fig 7G** revealed that Y-27632 and BA synergistically improved the distribution of ROCK2 and MLCK in LPS-induced Caco2 cells, while RhoA localization in Caco2 cells was similar to the condition in the absence of Y-27632. These data suggest that BA may improve the epithelial barrier function of Caco2 cells by inhibiting the RhoA/ROCK2/MLCK pathway, particularly by influencing the expression and localization of ROCK2 and MLCK in LPS-induced Caco2 cells.

## 4. Discussion

Numerous enteric and systemic diseases result in damage to the intestinal epithelial barrier. The pathogenesis of and potential treatments for such damage have been extensively studied [16]. However, there is an urgent need to identify effective techniques for utilizing food supplements that do not have adverse effects to reverse intestinal epithelial barrier damage and improve patient outcomes and prognosis. Consequently, identifying a new therapeutic regimen with the potential to alleviate intestinal epithelial barrier damage is a crucial focus of basic and clinical research. BA provides essential energy for colon cells and is vital for maintaining the integrity of the intestinal epithelium [17]. Numerous studies have established that LPS induces intestinal barrier damage, making it the standard reagent for modeling such intestinal barrier damage [10]. In this study, we employed LPS to develop an in vitro model of intestinal barrier damage. Additionally, the effect of BA, which can protect Caco2 cells on LPS-induced intestinal barrier damage and the associated underlying mechanisms were investigated. The present study demonstrated that BA increased cell viability, preserved the integrity and permeability of the intestinal epithelial barrier, upregulated the expression of ZO-1 and Occludin, maintained the subcellular localization of these proteins, and inhibited the RhoA/ROCK2/MLCK signaling pathway. Furthermore, BA partly reversed the changes in the intracellular distribution of RhoA, ROCK2, and MLCK, thereby, mitigating the damage caused by LPS on the intestinal barrier. Notably, the addition of Y-27632 further enhanced the epithelial barrier function when Y-27632 was combined with BA.

The intestinal mucosal barrier, which is composed of intestinal epithelial cells and mucus within the intestinal cavity, serves as the primary line of defense against pathogens [18]. The efficacy of this barrier is intrinsically linked to the integrity of the junctions between epithelial cells, particularly the tight junctions located on the apical surface. These tight junctions are composed of various proteins, including transmembrane proteins, adhesion molecules, and triglycerides. The transmembrane proteins include claudins, Occludins, and others, whereas the perimembrane proteins primarily include ZOs, such as ZO-1, ZO-2, and ZO-3. These proteins are crucial in linking the membrane proteins to the actin cytoskeleton and facilitating signal transduction. Furthermore, tight junctions are instrumental in regulating the movement of substances, such as ions and water, across the epithelial layer, thereby influencing transcellular permeability [19].

BA is a vital short-chain fatty acid that supplies the majority of the energy required by intestinal mucosal epithelial cells. The highest concentrations of BA are found in the colon, ranging from 10 to 20 mM, are found in the colon. Because up to 95% of BA is rapidly taken up and consumed locally in the gut as energy by the mucosa, the level of BA significantly decreases from the gut lumen to the feces (3.5 to 32.6 g/kg), then to the portal vein (~18 μmol/l) and subsequently to the peripheral blood **(**~20% of the portal vein concentrations) [20]. In a study in which 100 mg/kg sodium butyrate was administered orally daily, BA reduced gut inflammation and ameliorated symptoms of colitis in mice in a dose-dependent manner [21]. Furthermore, numerous studies have indicated that BA effectively enhances intestinal barrier integrity by promoting the expression of Claudin 2 and reducing intestinal permeability through an IL-10 receptor-dependent mechanism [22]. Additionally, BA has been demonstrated to activate genes that encode tight junction proteins and increase the expression of these proteins through a recombinant transcription factor, greatly improving intestinal epithelial barrier function when stabilized by hypoxia-inducible factor-1 [23]. Additionally, BA was reported to contribute to maintaining intestinal homeostasis [24]. Interestingly, treatment with low final concentrations (0–0.5 mmol/L) of BA has been shown to significantly increase cell viability, whereas higher concentrations of BA have an adverse effect. Specifically, the application of 0.2 mmol/L BA applied to Caco2 cells for 24 h was particularly effective at increasing cell viability, highlighting the protective effect of low-concentration BA on intestinal maintenance. Consequently, this study administered varying concentrations of LPS to Caco2 cells over different periods to determine the optimal concentration and duration of LPS intervention on the intestinal epithelial barrier. These results are consistent with those of previous studies [25]. The concentration effect of BA may be influenced by various factors, including the duration of the experiment and the cell culture conditions, and so on. The specific concentration selection should be selected according to the experimental purpose, preliminary results, and the existing literature.

In healthy individuals, the physiological concentration of LPS is typically very low, generally less than 1 endotoxin unit (EU)/mL. However, certain conditions, such as impaired intestinal barrier function or bacterial infections, can lead to elevated levels of LPS. Due to the existence of various LPS serotypes, and the use of different dosages in multiple studies, there is considerable variability in reported LPS concentrations [11, 26]. Consequently, in this study, varying concentrations of LPS were administered to Caco2 cells for different periods to determine the optimal concentration and treatment duration for LPS. These findings indicated that at LPS concentrations between 2 and 10 μg/mL, BA played a protective role in maintaining the epithelial barrier. Conversely, at concentrations exceeding 10 μg/mL, cell viability decreased rapidly. Therefore, an in vitro model was established by treating cells with 0.2 mmol/L BA and 5 μg/mL LPS for 24 h. In a separate study, a decrease in cell viability and an increase in corneal epithelial cell (CEC) cytotoxicity were observed when comparing the medium control to concentrations greater than 1 ng/mL of TNFα. Butyrate at a concentration of 5 mM and above reduced cell viability, whereas BA treatment alone (up to 25 mM) did not induce cytotoxicity compared to untreated cells. When CECs were co-treated with 500 μM BA and 1 ng/mL TNFα, the co-treatment with BA had a protective effect on cell viability and cytotoxicity compared with 1 ng/mL TNFα alone. These results suggest that BA exerts dose-specific protective effects against TNFα-induced reductions in the cell viability and cytotoxicity of rodent CECs. The observed efficacy of BA in mitigating LPS-induced injury in Caco2 epithelial cells, as demonstrated in this study, is consistent with findings in the existing literature [27]. Moreover, LPS was observed to decrease TEER, increase FD-4 flux, downregulate the expression of ZO-1 and Occludin, and alter their intracellular distribution. These findings align with those of previous studies [28, 29], suggesting that LPS induces dysfunction of the intestinal epithelial barrier. However, the administration of BA significantly mitigated these effects. Furthermore, a recent study demonstrated that the intake of BA led to a substantial up-regulation of transcripts encoding claudin-1, claudin-2, occludin, junctional adhesion molecule 3, and ZO-1 by RT-qPCR [30]. These results indicate that BA improves epithelial barrier integrity, which is consistent with earlier studies [9]. Our findings corroborate those of previous studies investigating the beneficial effects of BA on cellular viability and the modulation of tight junction protein expression in epithelial cells. Notably, the increase in cell viability following LPS-induced injury by BA is dependent on both the exposure time and concentration. Consequently, this investigation supports the hypothesis that BA primarily exerts its protective effects by increasing the expression of tight junction proteins between epithelial cells.

RhoA is a protein that plays important roles in the formation and function of cell junctions. However, the inhibition of RhoA can lead to abnormal tight junction function in epithelial cells [31]. Studies have shown that EphB2-Exos can protect cells and increase cell proliferation and migration by inhibiting the RhoA/ROCK pathway [32]. Inhibiting RhoA, ROCK1, and ROCK2 through the use of extracellular enzymes such as C3, Y-27632, and selective siRNAs significantly impairs epithelial cell migration [33]. Conversely, LPS and TNF-α can activate ROCK, resulting in the mislocalization of ZO-1 and claudin-2, which subsequently downregulates TEER and upregulates FD-4 flux in Caco2 cells [34]. Brain capillary endothelial cells are also affected by LPS exposure, leading to decreased cell viability, reduced TEER, and increased fluorescence yellow permeability. In both scenarios, the integrity of cell tight junctions is compromised, resulting in decreased expression of claudin-5 and ZO-1, as well as reduced mRNA expression of claudin-5 and Occludin. However, inhibiting of the RhoA/ROCK2 pathway via catalysis can reverse these effects and restore the expression of proteins such as ZO-1 and Occludin [35]. Additionally, inflammatory stimulation can activate MLCK and induce the endocytosis of Occludin, resulting in increased paracellular permeability [36]. LPS induces alterations in the distribution of ZO-1 and claudin-1 and activates the MLCK/MLC pathway, ultimately leading to increased permeability [37]. Sodium fluoride triggers the calcium-dependent RhoA/ROCK pathway, which compromises the integrity of the intestinal mucosa by disrupting the intracellular arrangement of ZO-1 and F-actin. MLCK promotes the disruption of ZO-1 in Caco2 cells, which further deteriorates their condition. Notably, Y-27632 has been found to reverse the effects of sodium fluoride [38]. Furthermore, a separate study demonstrated that LPS increases the mRNA expression of RhoA [39], ROCK2 [40], and MLCK [41], leading to alterations in their distribution within Caco2 cells. In these studies, researchers focused on BA and Caco2 cells, some focused on tight junctions and RhoA/ROCK2 or MLCK, and some reported changes in tight junctions in Caco2 cells, and so on. However, no study has focused on involvement of the RhoA/ROCK2/MLCK pathway in the protective effects of BA in Caco2 cells. In this study, LPS was observed to increase the expression of RhoA, ROCK2, and MLCK, thereby affecting the distribution of these proteins in Caco2 cells. RhoA is predominately located in the cell membrane and cytoplasm [42], whereas ROCK2 and MLCK are primarily localized in the cytoplasm, which is consistent with previous reports [43]. The expression of RhoA was significantly increased and translocated to the nucleus, however the expression of ROCK2 around the cytoplasm and membrane also significantly increased. Additionally, the expression levels of MLCK around the cell membrane and nucleus were notably elevated following LPS stimulation [44]. In contrast, after BA treatment, the expression of these proteins decreased and the change in their distribution was partially reversed [45]. Furthermore, when Y-27632 was administrated to Caco2 cells for 24 h, increase in cell viability, barrier integrity, and the expression and localization of ZO-1 and Occludin, along with enhanced activation of ROCK2 and MLCK were observed. However, the expression and localization of RhoA were comparable to those observed in the absence of Y-27632 [46]. In summary, Y-27632 exerted a synergistic effect with BA, further supporting the involvement of the RhoA/ROCK/MLCK pathway in the protective effect of BA against LPS-induced damage to the epithelial barrier.

This investigation has several limitations that warrant consideration. First, we did not assess the effects of RhoA and MLCK inhibitors [47], nor did we perform additional studies utilizing agonists of RhoA, ROCK2, and MLCK. Consequently, further research is needed to investigate the role of the RhoA/ROCK2/MLCK signaling pathway in the effect of BA using agonists and other inhibitors other than Y-27632. Second, our study focused solely on one signaling pathway, highlighting the need to broaden our experiments to encompass additional mechanisms. Third, the current study was exclusively conducted on Caco2 cells, thus, further studies on various intestinal epithelial cell lines are necessary. Fourth, while we robustly demonstrated that BA mitigates barrier impairment in epithelial cells following LPS injury through the up-regulation of ZO-1 and Occludin, elucidating the ability of BA to significantly increase cell viability is crucial. To clarify the relative importance of these effects, further experiments are needed. Such efforts will foster a deeper understanding of the complex mechanisms by which BA influences epithelial barrier function and cell resilience. Finally, we recommend adopting more precise and versatile molecular research methods to investigate BA’s direct effects on the RhoA/ROCK2/MLCK signaling pathway.

## 5. Conclusions

In summary, the data indicate that BA plays a crucial role in preserving the integrity of the intestinal barrier by increasing the viability of intestinal epithelial cells. Specifically, BA increases TEER, reduces FD-4 flux, and maintains barrier integrity. Furthermore, BA reversed the changes in the expression and localization of ZO-1 and Occludin in LPS-induced Caco2 cells. These effects are mediated through the activation of the RhoA/ROCK2/MLCK signaling pathway. This study enhances our understanding of the pathogenesis underlying LPS-induced intestinal injury and suggests that BA may represent a promising therapeutic option for patients experiencing intestinal barrier dysfunction.

## Supporting information

S1 File (ZIP)
