# Supplementary figures and images for: Butyric acid alleviates LPS-induced intestinal mucosal barrier damage by inhibiting the RhoA/ROCK2/MLCK signaling pathway in Caco2 cells (part 2 of 2)
Source: PLoS One. 2024 Dec 26;19(12):e0316362. doi: 10.1371/journal.pone.0316362 (PMC11670954; doi:10.1371/journal.pone.0316362)

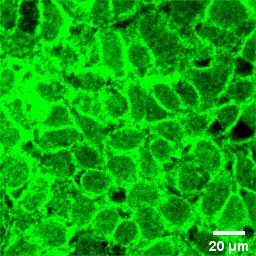

Supplement: S1 File — (ZIP) [file pone.0316362.s001.zip › PLOS ONE Surporting infomation/raw image/Fig 7G/C2-LI group MLCK_20x1_RGB(1).jpg]

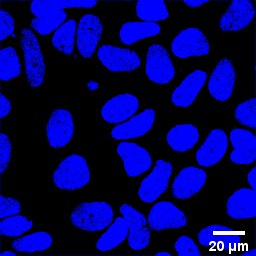

Supplement: S1 File — (ZIP) [file pone.0316362.s001.zip › PLOS ONE Surporting infomation/raw image/Fig 7G/C3-BI group MLCK_20x3_RGB(1).jpg]

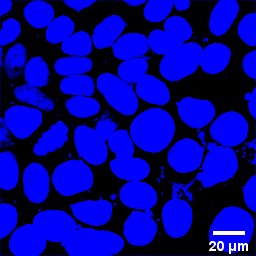

Supplement: S1 File — (ZIP) [file pone.0316362.s001.zip › PLOS ONE Surporting infomation/raw image/Fig 7G/C3-BI group RhoA_20x9_RGB(1)蓝色.jpg]

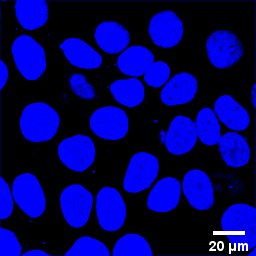

Supplement: S1 File — (ZIP) [file pone.0316362.s001.zip › PLOS ONE Surporting infomation/raw image/Fig 7G/C3-BI group ROCK_20x8_RGB(1).jpg]

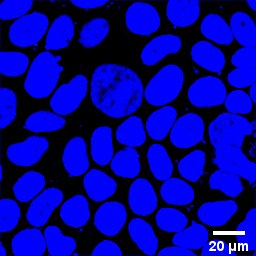

Supplement: S1 File — (ZIP) [file pone.0316362.s001.zip › PLOS ONE Surporting infomation/raw image/Fig 7G/C3-BLI group RhoA_20x2_RGB(1)蓝色.jpg]

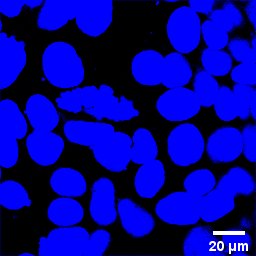

Supplement: S1 File — (ZIP) [file pone.0316362.s001.zip › PLOS ONE Surporting infomation/raw image/Fig 7G/C3-BLI group ROCK_20x5_RGB(1).jpg]

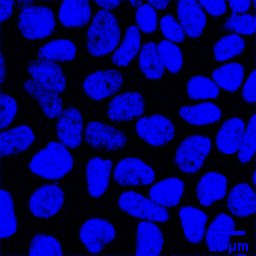

Supplement: S1 File — (ZIP) [file pone.0316362.s001.zip › PLOS ONE Surporting infomation/raw image/Fig 7G/C3-BLI MLCK group 20x10.jpg]

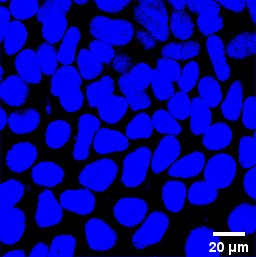

Supplement: S1 File — (ZIP) [file pone.0316362.s001.zip › PLOS ONE Surporting infomation/raw image/Fig 7G/C3-CI group MLCK_20x12_RGB(1).jpg]

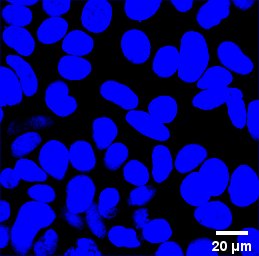

Supplement: S1 File — (ZIP) [file pone.0316362.s001.zip › PLOS ONE Surporting infomation/raw image/Fig 7G/C3-CI group ROCK_20x4_RGB(1)蓝色.jpg]

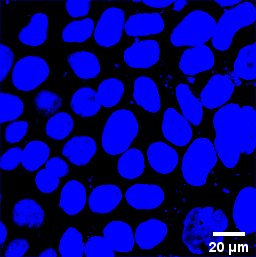

Supplement: S1 File — (ZIP) [file pone.0316362.s001.zip › PLOS ONE Surporting infomation/raw image/Fig 7G/C3-CI group RhoA_20x19_RGB(1)蓝色.jpg]

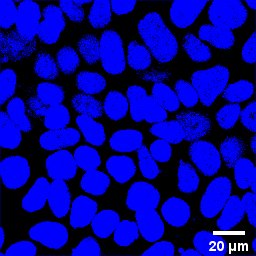

Supplement: S1 File — (ZIP) [file pone.0316362.s001.zip › PLOS ONE Surporting infomation/raw image/Fig 7G/C3-LI group MLCK_20x1_RGB(1).jpg]

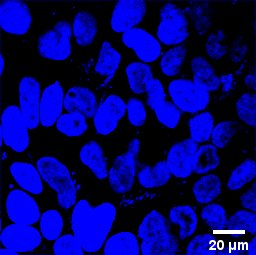

Supplement: S1 File — (ZIP) [file pone.0316362.s001.zip › PLOS ONE Surporting infomation/raw image/Fig 7G/C3-LI group RhoA_20x36_RGB蓝色.jpg]

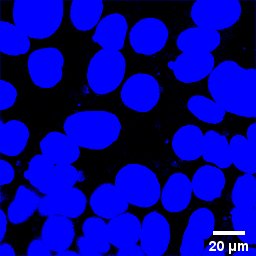

Supplement: S1 File — (ZIP) [file pone.0316362.s001.zip › PLOS ONE Surporting infomation/raw image/Fig 7G/C3-LI group ROCK_20x1_RGB(1)蓝色.jpg]

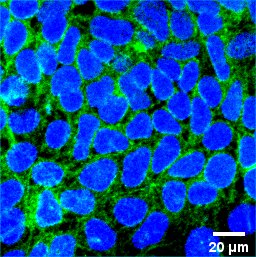

Supplement: S1 File — (ZIP) [file pone.0316362.s001.zip › PLOS ONE Surporting infomation/raw image/Fig 7G/CI group MLCK 20x12合并.jpg]

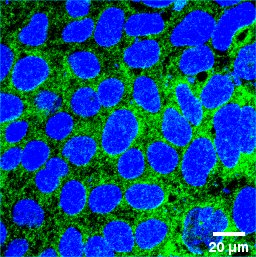

Supplement: S1 File — (ZIP) [file pone.0316362.s001.zip › PLOS ONE Surporting infomation/raw image/Fig 7G/CI group RhoA 20x19合并.jpg]

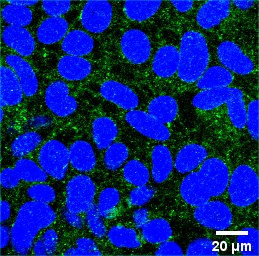

Supplement: S1 File — (ZIP) [file pone.0316362.s001.zip › PLOS ONE Surporting infomation/raw image/Fig 7G/CI group ROCK 20x4.jpg]

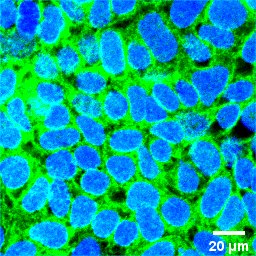

Supplement: S1 File — (ZIP) [file pone.0316362.s001.zip › PLOS ONE Surporting infomation/raw image/Fig 7G/LI group MLCK 20x1.jpg]

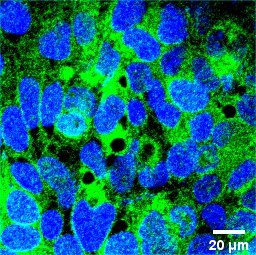

Supplement: S1 File — (ZIP) [file pone.0316362.s001.zip › PLOS ONE Surporting infomation/raw image/Fig 7G/LI group RhoA_20x36_RGB.jpg]

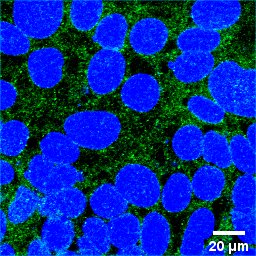

Supplement: S1 File — (ZIP) [file pone.0316362.s001.zip › PLOS ONE Surporting infomation/raw image/Fig 7G/LI group ROCK 20x1合并.jpg]
