# Supplementary figures and images for: Butyric acid alleviates LPS-induced intestinal mucosal barrier damage by inhibiting the RhoA/ROCK2/MLCK signaling pathway in Caco2 cells (part 1 of 2)
Source: PLoS One. 2024 Dec 26;19(12):e0316362. doi: 10.1371/journal.pone.0316362 (PMC11670954; doi:10.1371/journal.pone.0316362)

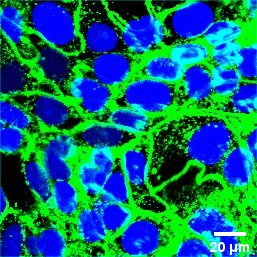

Supplement: S1 File — (ZIP) [file pone.0316362.s001.zip › PLOS ONE Surporting infomation/raw image/Fig 3E/BA group Occludin 20x4合并.jpg]

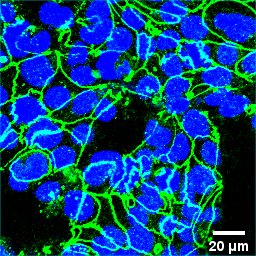

Supplement: S1 File — (ZIP) [file pone.0316362.s001.zip › PLOS ONE Surporting infomation/raw image/Fig 3E/BA group ZO-1 20x4.jpg]

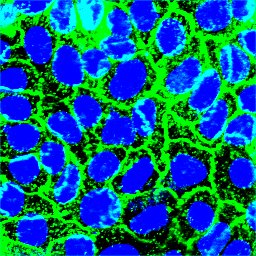

Supplement: S1 File — (ZIP) [file pone.0316362.s001.zip › PLOS ONE Surporting infomation/raw image/Fig 3E/BL group Occludin 20x3合并.jpg]

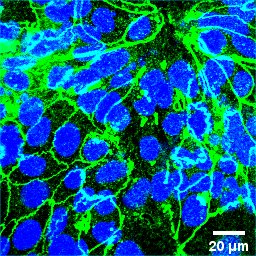

Supplement: S1 File — (ZIP) [file pone.0316362.s001.zip › PLOS ONE Surporting infomation/raw image/Fig 3E/BL group ZO-1 20x3.jpg]

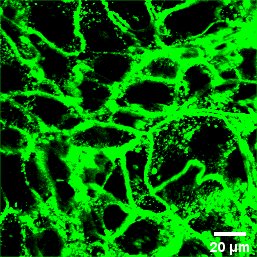

Supplement: S1 File — (ZIP) [file pone.0316362.s001.zip › PLOS ONE Surporting infomation/raw image/Fig 3E/C2-BA group Occludin_20x4_RGB(1)绿色.jpg]

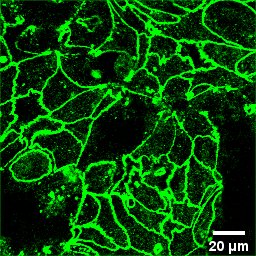

Supplement: S1 File — (ZIP) [file pone.0316362.s001.zip › PLOS ONE Surporting infomation/raw image/Fig 3E/C2-BA group ZO-1_20x4_RGB(1).jpg]

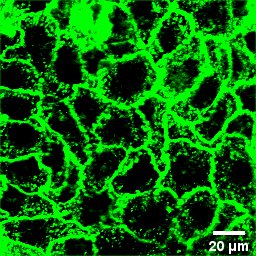

Supplement: S1 File — (ZIP) [file pone.0316362.s001.zip › PLOS ONE Surporting infomation/raw image/Fig 3E/C2-BL group Occludin_20x3_RGB(1).jpg]

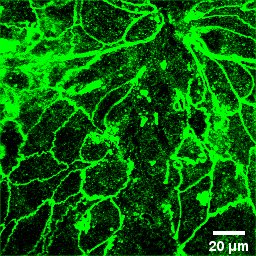

Supplement: S1 File — (ZIP) [file pone.0316362.s001.zip › PLOS ONE Surporting infomation/raw image/Fig 3E/C2-BL group ZO-1_20x3_RGB(1).jpg]

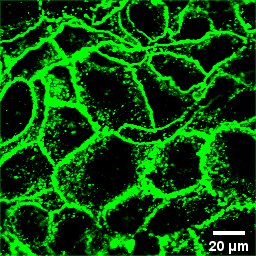

Supplement: S1 File — (ZIP) [file pone.0316362.s001.zip › PLOS ONE Surporting infomation/raw image/Fig 3E/C2-control group Occludin_20x11_RGB 绿色.jpg]

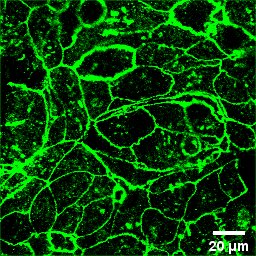

Supplement: S1 File — (ZIP) [file pone.0316362.s001.zip › PLOS ONE Surporting infomation/raw image/Fig 3E/C2-control group ZO-1_20x4_RGB.jpg]

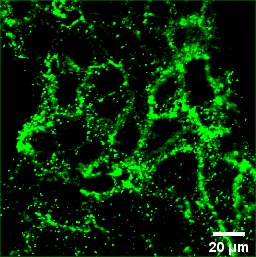

Supplement: S1 File — (ZIP) [file pone.0316362.s001.zip › PLOS ONE Surporting infomation/raw image/Fig 3E/C2-LPS group Occludin_20x19_RGB(1)绿色.jpg]

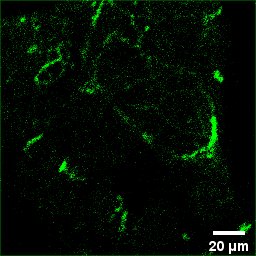

Supplement: S1 File — (ZIP) [file pone.0316362.s001.zip › PLOS ONE Surporting infomation/raw image/Fig 3E/C2-LPS group ZO-1_20x1_RGB(1).jpg]

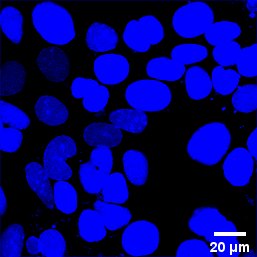

Supplement: S1 File — (ZIP) [file pone.0316362.s001.zip › PLOS ONE Surporting infomation/raw image/Fig 3E/C3-BA group Occludin_20x4_RGB(1)l蓝色.jpg]

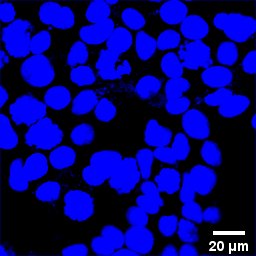

Supplement: S1 File — (ZIP) [file pone.0316362.s001.zip › PLOS ONE Surporting infomation/raw image/Fig 3E/C3-BA group ZO-1_20x4_RGB(1).jpg]

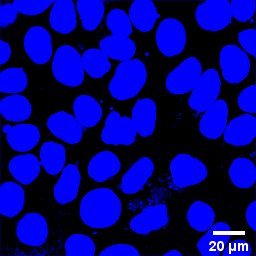

Supplement: S1 File — (ZIP) [file pone.0316362.s001.zip › PLOS ONE Surporting infomation/raw image/Fig 3E/C3-BL group Occludin_20x3_RGB(1)蓝色.jpg]

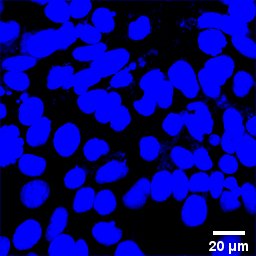

Supplement: S1 File — (ZIP) [file pone.0316362.s001.zip › PLOS ONE Surporting infomation/raw image/Fig 3E/C3-BL group ZO-1_20x3_RGB(1).jpg]

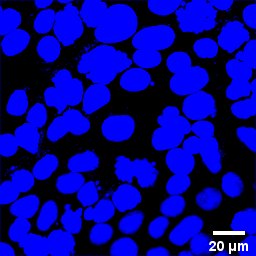

Supplement: S1 File — (ZIP) [file pone.0316362.s001.zip › PLOS ONE Surporting infomation/raw image/Fig 3E/C3-control goup ZO-1_20x4_RGB.jpg]

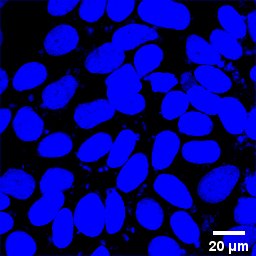

Supplement: S1 File — (ZIP) [file pone.0316362.s001.zip › PLOS ONE Surporting infomation/raw image/Fig 3E/C3-control group Occludin_20x11_RGB(1)蓝色.jpg]

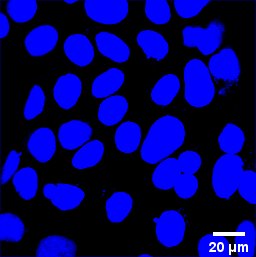

Supplement: S1 File — (ZIP) [file pone.0316362.s001.zip › PLOS ONE Surporting infomation/raw image/Fig 3E/C3-LPS group Occludin_20x19_RGB(1)蓝色.jpg]

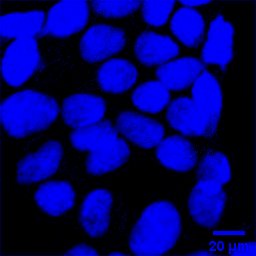

Supplement: S1 File — (ZIP) [file pone.0316362.s001.zip › PLOS ONE Surporting infomation/raw image/Fig 3E/C3-LPS group ZO-1 20x6.jpg]

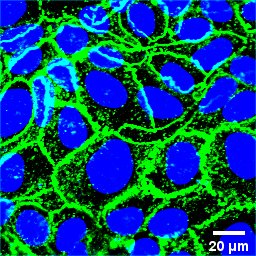

Supplement: S1 File — (ZIP) [file pone.0316362.s001.zip › PLOS ONE Surporting infomation/raw image/Fig 3E/control group Occludin 20x11合并.jpg]

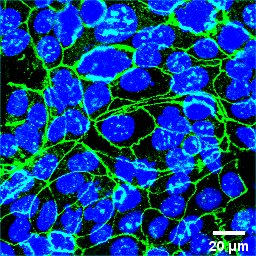

Supplement: S1 File — (ZIP) [file pone.0316362.s001.zip › PLOS ONE Surporting infomation/raw image/Fig 3E/control group ZO-1_20x4_RGB.jpg]

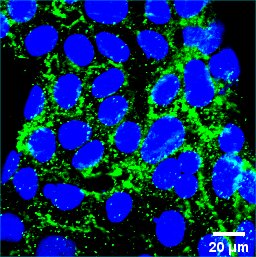

Supplement: S1 File — (ZIP) [file pone.0316362.s001.zip › PLOS ONE Surporting infomation/raw image/Fig 3E/LPS group Occludin 20x19 合并.jpg]

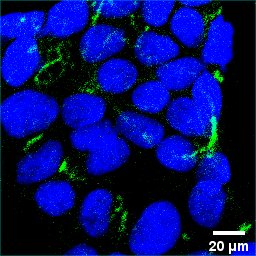

Supplement: S1 File — (ZIP) [file pone.0316362.s001.zip › PLOS ONE Surporting infomation/raw image/Fig 3E/LPS group ZO-1 20x6.jpg]

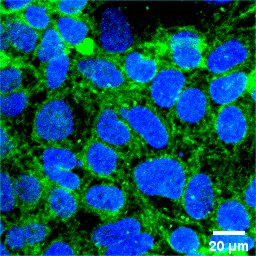

Supplement: S1 File — (ZIP) [file pone.0316362.s001.zip › PLOS ONE Surporting infomation/raw image/Fig 4G/BA group MLCK 20x9.jpg]

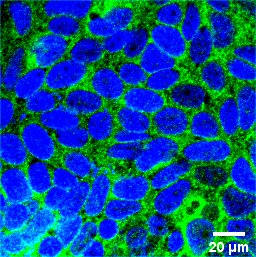

Supplement: S1 File — (ZIP) [file pone.0316362.s001.zip › PLOS ONE Surporting infomation/raw image/Fig 4G/BA group RhoA 20x2 合并.jpg]

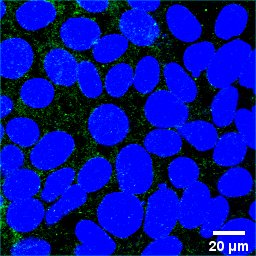

Supplement: S1 File — (ZIP) [file pone.0316362.s001.zip › PLOS ONE Surporting infomation/raw image/Fig 4G/BA group ROCK 20x9合并.jpg]

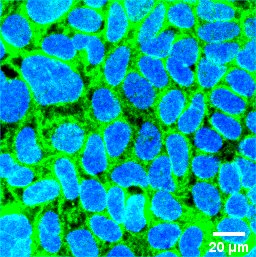

Supplement: S1 File — (ZIP) [file pone.0316362.s001.zip › PLOS ONE Surporting infomation/raw image/Fig 4G/BL group MLCK 20x4.jpg]

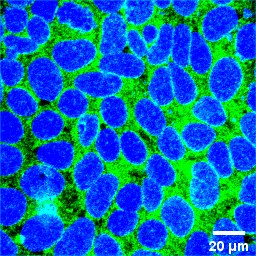

Supplement: S1 File — (ZIP) [file pone.0316362.s001.zip › PLOS ONE Surporting infomation/raw image/Fig 4G/BL group RhoA 20x6 合并.jpg]

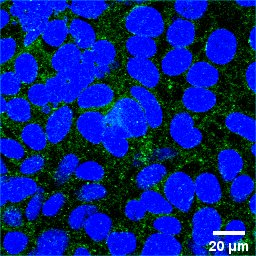

Supplement: S1 File — (ZIP) [file pone.0316362.s001.zip › PLOS ONE Surporting infomation/raw image/Fig 4G/BLgroup ROCK 20x7.jpg]

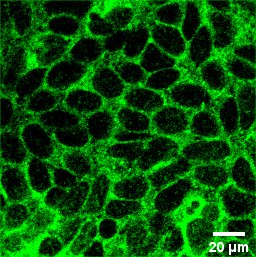

Supplement: S1 File — (ZIP) [file pone.0316362.s001.zip › PLOS ONE Surporting infomation/raw image/Fig 4G/C2-BA group RhoA_20x2_RGB绿色.jpg]

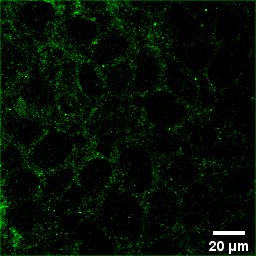

Supplement: S1 File — (ZIP) [file pone.0316362.s001.zip › PLOS ONE Surporting infomation/raw image/Fig 4G/C2-BA group ROCK_20x9_RGB(1).jpg]

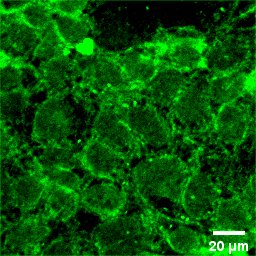

Supplement: S1 File — (ZIP) [file pone.0316362.s001.zip › PLOS ONE Surporting infomation/raw image/Fig 4G/C2-BA group MLCK_20x9_RGB(1).jpg]

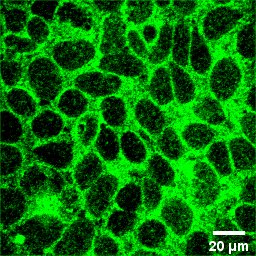

Supplement: S1 File — (ZIP) [file pone.0316362.s001.zip › PLOS ONE Surporting infomation/raw image/Fig 4G/C2-BL group RhoA_20x6_RGB(1)绿色.jpg]

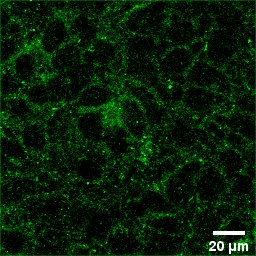

Supplement: S1 File — (ZIP) [file pone.0316362.s001.zip › PLOS ONE Surporting infomation/raw image/Fig 4G/C2-BL group ROCK_20x7_RGB(1).jpg]

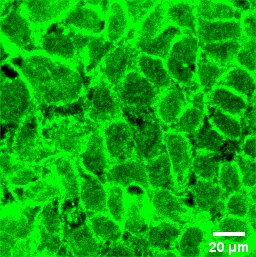

Supplement: S1 File — (ZIP) [file pone.0316362.s001.zip › PLOS ONE Surporting infomation/raw image/Fig 4G/C2-BL group MLCK_20x4_RGB(1).jpg]

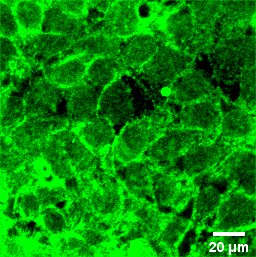

Supplement: S1 File — (ZIP) [file pone.0316362.s001.zip › PLOS ONE Surporting infomation/raw image/Fig 4G/C2-control group MLCK_20x7_RGB(1).jpg]

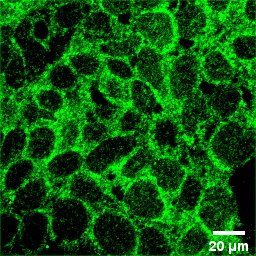

Supplement: S1 File — (ZIP) [file pone.0316362.s001.zip › PLOS ONE Surporting infomation/raw image/Fig 4G/C2-control group RhoA_20x23_RGB(2)绿色.jpg]

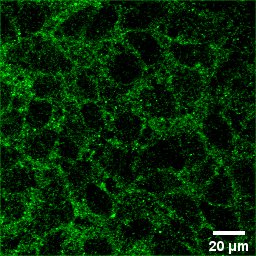

Supplement: S1 File — (ZIP) [file pone.0316362.s001.zip › PLOS ONE Surporting infomation/raw image/Fig 4G/C2-control group ROCK_20x7_RGB(1)绿色.jpg]

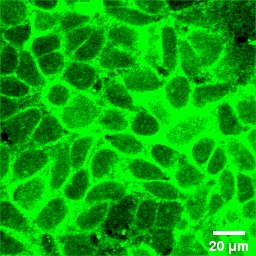

Supplement: S1 File — (ZIP) [file pone.0316362.s001.zip › PLOS ONE Surporting infomation/raw image/Fig 4G/C2-LPS group MLCK_20x3_RGB(1).jpg]

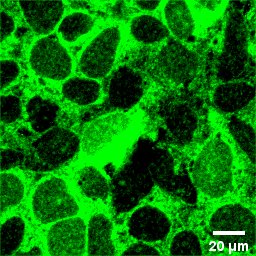

Supplement: S1 File — (ZIP) [file pone.0316362.s001.zip › PLOS ONE Surporting infomation/raw image/Fig 4G/C2-LPS group RhoA_20x26_RGB(1)绿色.jpg]

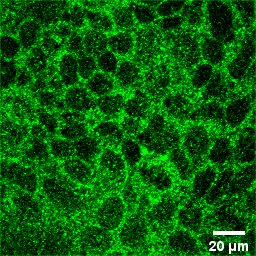

Supplement: S1 File — (ZIP) [file pone.0316362.s001.zip › PLOS ONE Surporting infomation/raw image/Fig 4G/C2-LPS group ROCK_20x1_RGB(1).jpg]

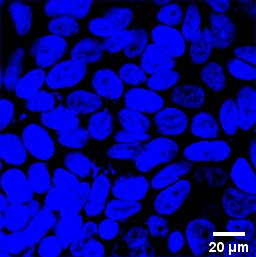

Supplement: S1 File — (ZIP) [file pone.0316362.s001.zip › PLOS ONE Surporting infomation/raw image/Fig 4G/C3-BA group RhoA_20x2_RGB蓝色.jpg]

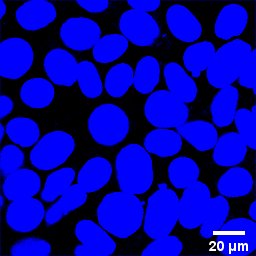

Supplement: S1 File — (ZIP) [file pone.0316362.s001.zip › PLOS ONE Surporting infomation/raw image/Fig 4G/C3-BA group ROCK_20x9_RGB(1).jpg]

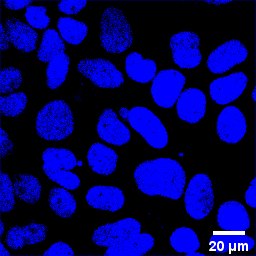

Supplement: S1 File — (ZIP) [file pone.0316362.s001.zip › PLOS ONE Surporting infomation/raw image/Fig 4G/C3-BA group MLCK_20x9_RGB(1).jpg]

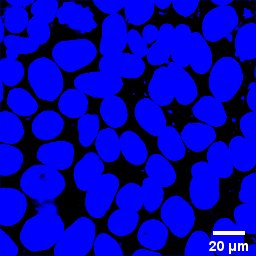

Supplement: S1 File — (ZIP) [file pone.0316362.s001.zip › PLOS ONE Surporting infomation/raw image/Fig 4G/C3-BL group RhoA_20x6_RGB(1)蓝色.jpg]

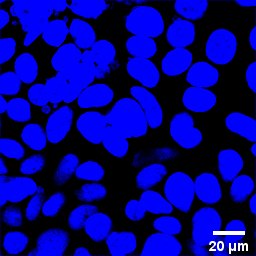

Supplement: S1 File — (ZIP) [file pone.0316362.s001.zip › PLOS ONE Surporting infomation/raw image/Fig 4G/C3-BL group ROCK_20x7_RGB(1).jpg]

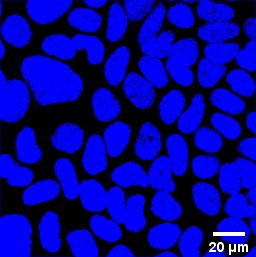

Supplement: S1 File — (ZIP) [file pone.0316362.s001.zip › PLOS ONE Surporting infomation/raw image/Fig 4G/C3-BL group MLCK_20x4_RGB(1).jpg]

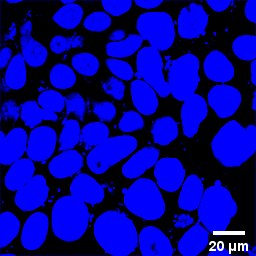

Supplement: S1 File — (ZIP) [file pone.0316362.s001.zip › PLOS ONE Surporting infomation/raw image/Fig 4G/C3-control group RhoA_20x23_RGB(2)蓝色.jpg]

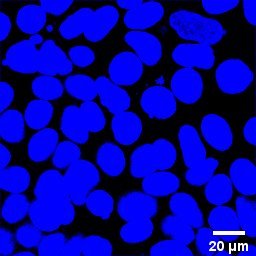

Supplement: S1 File — (ZIP) [file pone.0316362.s001.zip › PLOS ONE Surporting infomation/raw image/Fig 4G/C3-control group ROCK_20x7_RGB(1)蓝色.jpg]

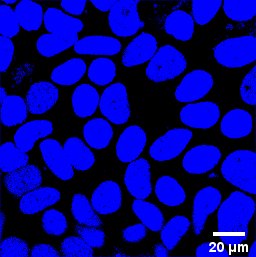

Supplement: S1 File — (ZIP) [file pone.0316362.s001.zip › PLOS ONE Surporting infomation/raw image/Fig 4G/C3-control group MLCK_20x7_RGB(1).jpg]

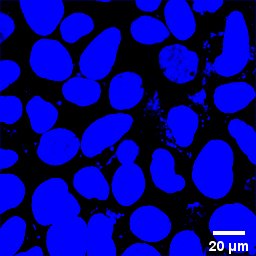

Supplement: S1 File — (ZIP) [file pone.0316362.s001.zip › PLOS ONE Surporting infomation/raw image/Fig 4G/C3-LPS group RhoA_20x26_RGB(1)蓝色.jpg]

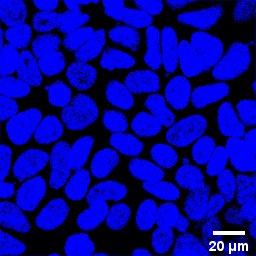

Supplement: S1 File — (ZIP) [file pone.0316362.s001.zip › PLOS ONE Surporting infomation/raw image/Fig 4G/C3-LPS group MLCK_20x3_RGB(1).jpg]

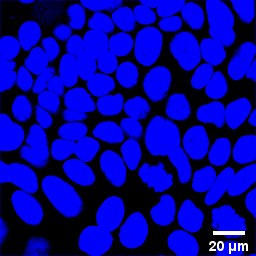

Supplement: S1 File — (ZIP) [file pone.0316362.s001.zip › PLOS ONE Surporting infomation/raw image/Fig 4G/C3-LPSgroup ROCK_20x1_RGB(1).jpg]

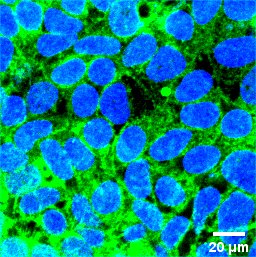

Supplement: S1 File — (ZIP) [file pone.0316362.s001.zip › PLOS ONE Surporting infomation/raw image/Fig 4G/control group MLCK 20x7.jpg]

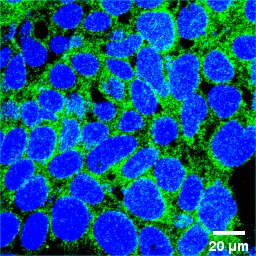

Supplement: S1 File — (ZIP) [file pone.0316362.s001.zip › PLOS ONE Surporting infomation/raw image/Fig 4G/control group RhoA 20x23合并.jpg]

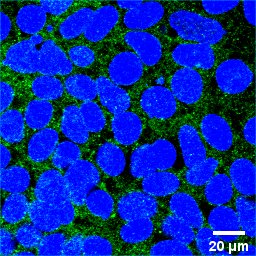

Supplement: S1 File — (ZIP) [file pone.0316362.s001.zip › PLOS ONE Surporting infomation/raw image/Fig 4G/control group ROCK 20x7合并.jpg]

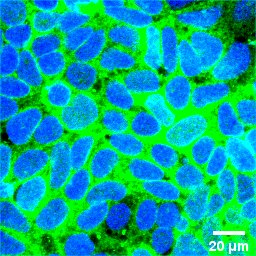

Supplement: S1 File — (ZIP) [file pone.0316362.s001.zip › PLOS ONE Surporting infomation/raw image/Fig 4G/LPS group MLCK 20x3.jpg]

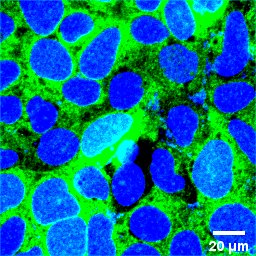

Supplement: S1 File — (ZIP) [file pone.0316362.s001.zip › PLOS ONE Surporting infomation/raw image/Fig 4G/LPS group RhoA 20x26 合并.jpg]

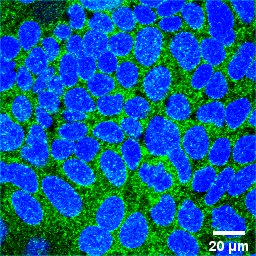

Supplement: S1 File — (ZIP) [file pone.0316362.s001.zip › PLOS ONE Surporting infomation/raw image/Fig 4G/LPS group ROCK 20x1.jpg]

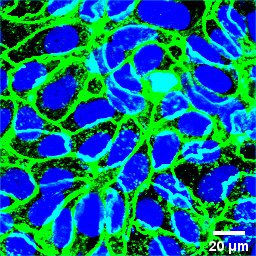

Supplement: S1 File — (ZIP) [file pone.0316362.s001.zip › PLOS ONE Surporting infomation/raw image/Fig 6E/BI group Occludin_20x11_RGB合并.jpg]

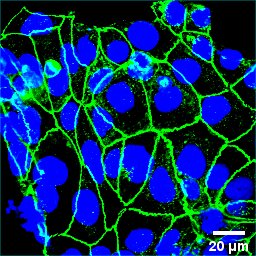

Supplement: S1 File — (ZIP) [file pone.0316362.s001.zip › PLOS ONE Surporting infomation/raw image/Fig 6E/BI group ZO-1 20x1.jpg]

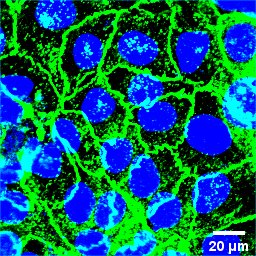

Supplement: S1 File — (ZIP) [file pone.0316362.s001.zip › PLOS ONE Surporting infomation/raw image/Fig 6E/BLI group Occludin 20x4合并1.jpg]

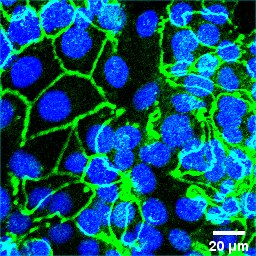

Supplement: S1 File — (ZIP) [file pone.0316362.s001.zip › PLOS ONE Surporting infomation/raw image/Fig 6E/BLI group ZO-1 20x1.jpg]

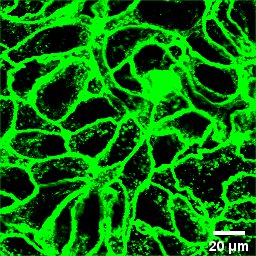

Supplement: S1 File — (ZIP) [file pone.0316362.s001.zip › PLOS ONE Surporting infomation/raw image/Fig 6E/C2-BI group Occludin_20x11_RGB(1)绿色.jpg]

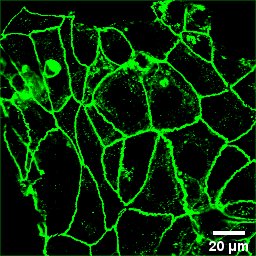

Supplement: S1 File — (ZIP) [file pone.0316362.s001.zip › PLOS ONE Surporting infomation/raw image/Fig 6E/C2-BI group ZO-1_20x1_RGB(1).jpg]

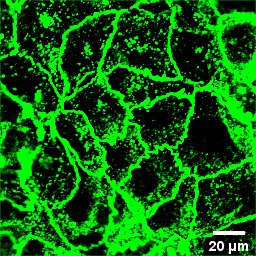

Supplement: S1 File — (ZIP) [file pone.0316362.s001.zip › PLOS ONE Surporting infomation/raw image/Fig 6E/C2-BLI group Occludin_20x4_RGB(1)绿色.jpg]

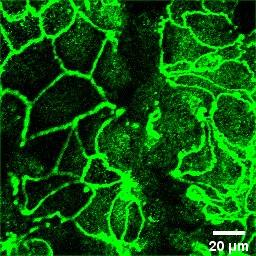

Supplement: S1 File — (ZIP) [file pone.0316362.s001.zip › PLOS ONE Surporting infomation/raw image/Fig 6E/C2-BLI group ZO-1_20x2_RGB(1).jpg]

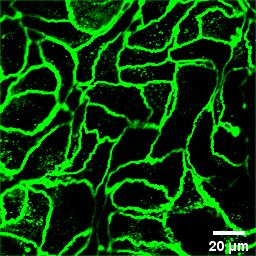

Supplement: S1 File — (ZIP) [file pone.0316362.s001.zip › PLOS ONE Surporting infomation/raw image/Fig 6E/C2-CI group Occludin_20x23_RGB(1)绿色.jpg]

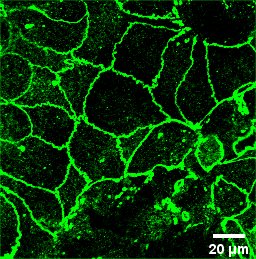

Supplement: S1 File — (ZIP) [file pone.0316362.s001.zip › PLOS ONE Surporting infomation/raw image/Fig 6E/C2-CI group ZO-1_20x5_RGB(1).jpg]

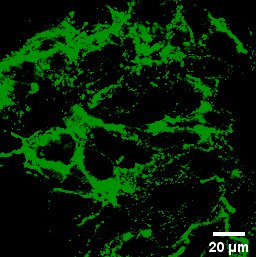

Supplement: S1 File — (ZIP) [file pone.0316362.s001.zip › PLOS ONE Surporting infomation/raw image/Fig 6E/C2-LPS group Occludin_20x13_RGB绿色.jpg]

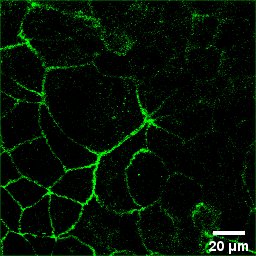

Supplement: S1 File — (ZIP) [file pone.0316362.s001.zip › PLOS ONE Surporting infomation/raw image/Fig 6E/C2-LPS group ZO-1_20x7_RGB(1).jpg]

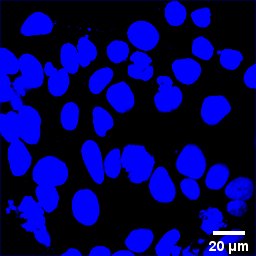

Supplement: S1 File — (ZIP) [file pone.0316362.s001.zip › PLOS ONE Surporting infomation/raw image/Fig 6E/C3-BI group ZO-1_20x1_RGB(1).jpg]

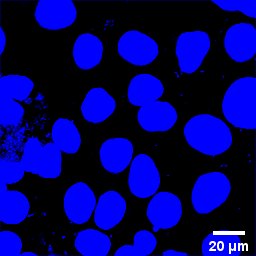

Supplement: S1 File — (ZIP) [file pone.0316362.s001.zip › PLOS ONE Surporting infomation/raw image/Fig 6E/C3-BLI group Occludin_20x4_RGB(1)蓝色.jpg]

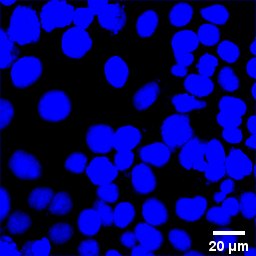

Supplement: S1 File — (ZIP) [file pone.0316362.s001.zip › PLOS ONE Surporting infomation/raw image/Fig 6E/C3-BLI group ZO-1_20x2_RGB(1).jpg]

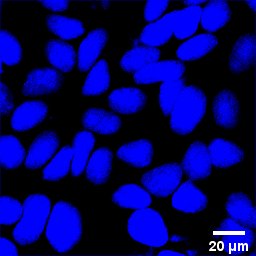

Supplement: S1 File — (ZIP) [file pone.0316362.s001.zip › PLOS ONE Surporting infomation/raw image/Fig 6E/C3-CI group Occludin_20x23_RGB(1)蓝色.jpg]

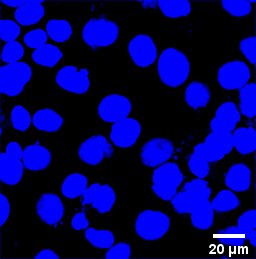

Supplement: S1 File — (ZIP) [file pone.0316362.s001.zip › PLOS ONE Surporting infomation/raw image/Fig 6E/C3-CI group ZO-1_20x5_RGB(1).jpg]

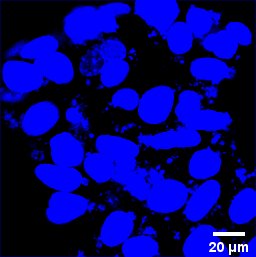

Supplement: S1 File — (ZIP) [file pone.0316362.s001.zip › PLOS ONE Surporting infomation/raw image/Fig 6E/C3-LPS group Occludin_20x13_RGB蓝色.jpg]

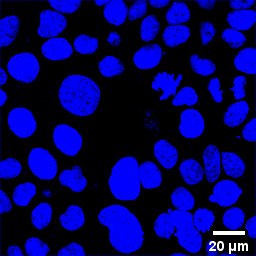

Supplement: S1 File — (ZIP) [file pone.0316362.s001.zip › PLOS ONE Surporting infomation/raw image/Fig 6E/C3-LPS group ZO-1_20x7_RGB(1).jpg]

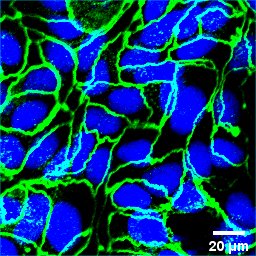

Supplement: S1 File — (ZIP) [file pone.0316362.s001.zip › PLOS ONE Surporting infomation/raw image/Fig 6E/CI group Occludin_20x23_RGB合并.jpg]

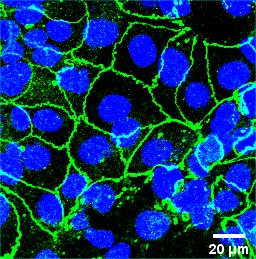

Supplement: S1 File — (ZIP) [file pone.0316362.s001.zip › PLOS ONE Surporting infomation/raw image/Fig 6E/CI group ZO-1 20x5.jpg]

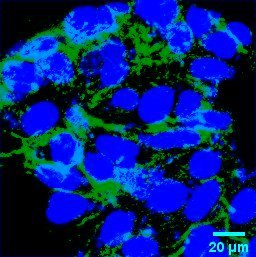

Supplement: S1 File — (ZIP) [file pone.0316362.s001.zip › PLOS ONE Surporting infomation/raw image/Fig 6E/LPS group Occludin_20x13_RGB合并.jpg]

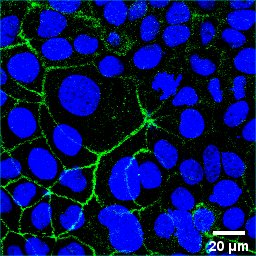

Supplement: S1 File — (ZIP) [file pone.0316362.s001.zip › PLOS ONE Surporting infomation/raw image/Fig 6E/LPS group ZO-1 20x7.jpg]

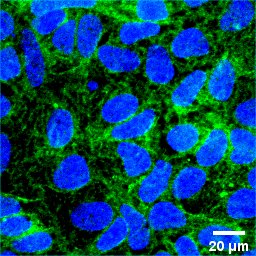

Supplement: S1 File — (ZIP) [file pone.0316362.s001.zip › PLOS ONE Surporting infomation/raw image/Fig 7G/BI group MLCK 20x3.jpg]

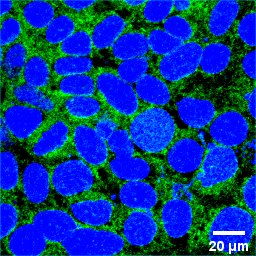

Supplement: S1 File — (ZIP) [file pone.0316362.s001.zip › PLOS ONE Surporting infomation/raw image/Fig 7G/BI group RhoA 20x9 合并.jpg]

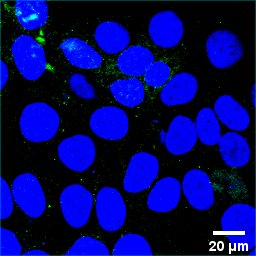

Supplement: S1 File — (ZIP) [file pone.0316362.s001.zip › PLOS ONE Surporting infomation/raw image/Fig 7G/BI group ROCK 20x8合并.jpg]

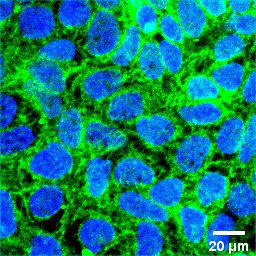

Supplement: S1 File — (ZIP) [file pone.0316362.s001.zip › PLOS ONE Surporting infomation/raw image/Fig 7G/BLI group MLCK 20x10.jpg]

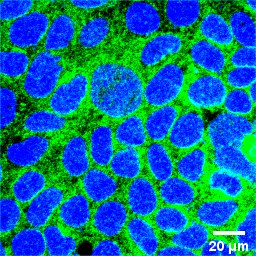

Supplement: S1 File — (ZIP) [file pone.0316362.s001.zip › PLOS ONE Surporting infomation/raw image/Fig 7G/BLI group RhoA 20x2合并.jpg]

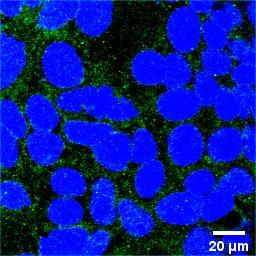

Supplement: S1 File — (ZIP) [file pone.0316362.s001.zip › PLOS ONE Surporting infomation/raw image/Fig 7G/BLI group ROCK 20x5合并.jpg]

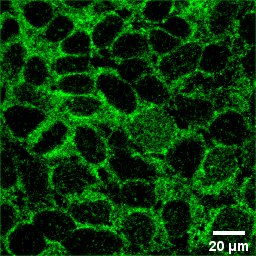

Supplement: S1 File — (ZIP) [file pone.0316362.s001.zip › PLOS ONE Surporting infomation/raw image/Fig 7G/C2-BI group RhoA_20x9_RGB(1)绿色.jpg]

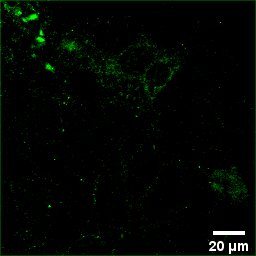

Supplement: S1 File — (ZIP) [file pone.0316362.s001.zip › PLOS ONE Surporting infomation/raw image/Fig 7G/C2-BI group ROCK_20x8_RGB(1).jpg]

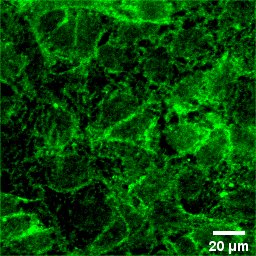

Supplement: S1 File — (ZIP) [file pone.0316362.s001.zip › PLOS ONE Surporting infomation/raw image/Fig 7G/C2-BI group MLCK_20x3_RGB(1).jpg]

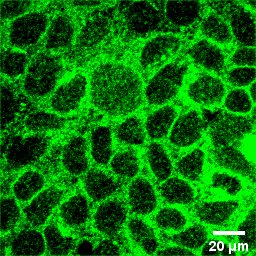

Supplement: S1 File — (ZIP) [file pone.0316362.s001.zip › PLOS ONE Surporting infomation/raw image/Fig 7G/C2-BLI group RhoA_20x2_RGB(1)绿色.jpg]

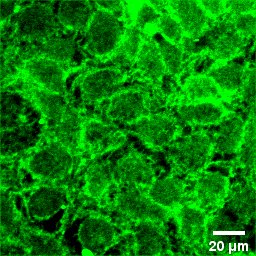

Supplement: S1 File — (ZIP) [file pone.0316362.s001.zip › PLOS ONE Surporting infomation/raw image/Fig 7G/C2-BLI group MLCK_20x10_RGB(1).jpg]

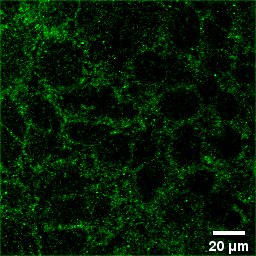

Supplement: S1 File — (ZIP) [file pone.0316362.s001.zip › PLOS ONE Surporting infomation/raw image/Fig 7G/C2-BLI group ROCK_20x5_RGB(1).jpg]

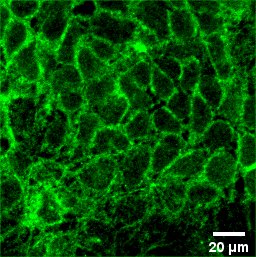

Supplement: S1 File — (ZIP) [file pone.0316362.s001.zip › PLOS ONE Surporting infomation/raw image/Fig 7G/C2-CI group MLCK_20x12_RGB(1).jpg]

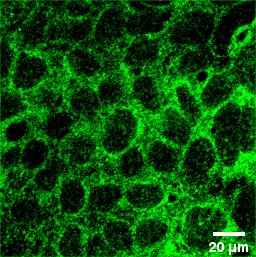

Supplement: S1 File — (ZIP) [file pone.0316362.s001.zip › PLOS ONE Surporting infomation/raw image/Fig 7G/C2-CI group RhoA_20x19_RGB(1)绿色.jpg]

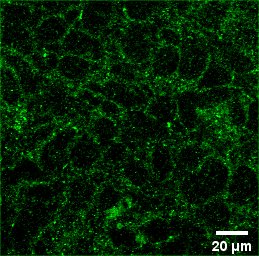

Supplement: S1 File — (ZIP) [file pone.0316362.s001.zip › PLOS ONE Surporting infomation/raw image/Fig 7G/C2-CI group ROCK_20x4_RGB(1).jpg]

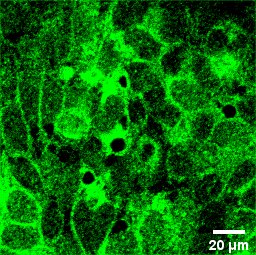

Supplement: S1 File — (ZIP) [file pone.0316362.s001.zip › PLOS ONE Surporting infomation/raw image/Fig 7G/C2-LI group RhoA_20x36_RGB绿色.jpg]

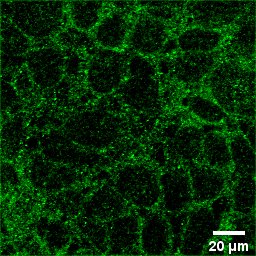

Supplement: S1 File — (ZIP) [file pone.0316362.s001.zip › PLOS ONE Surporting infomation/raw image/Fig 7G/C2-LI group ROCK_20x1_RGB(1)蓝色.jpg]
